# Anti-Depressant Fluoxetine Reveals its Therapeutic Effect Via Astrocytes

**DOI:** 10.1016/j.ebiom.2018.05.036

**Published:** 2018-06-08

**Authors:** Manao Kinoshita, Yuri Hirayama, Kayoko Fujishita, Keisuke Shibata, Youichi Shinozaki, Eiji Shigetomi, Akiko Takeda, Ha Pham Ngoc Le, Hideaki Hayashi, Miki Hiasa, Yoshinori Moriyama, Kazuhiro Ikenaka, Kenji F. Tanaka, Schuichi Koizumi

**Affiliations:** a Department of Neuropharmacology, Interdisciplinary Graduate School of Medicine, University of Yamanashi, Yamanashi 409-3898, Japan; b Department of Membrane Biochemistry, Okayama University, Graduate School of Medicine, Dentistry, and Pharmaceutical Sciences, Okayama, Japan; c Department of Biochemistry, Matsumoto Dental University, Shiojiri 399-0781, Japan; d Division of Neurobiology and Bioinformatics, National Institute for Physiological Sciences, Okazaki 444-8585, Japan; e Department of Neuropsychiatry, Keio University School of Medicine, Tokyo 160-8582, Japan

**Keywords:** Astrocytes, Fluoxetine, ATP, Adenosine, Vesicular nucleotide transporter, BDNF

## Abstract

Although psychotropic drugs act on neurons and glial cells, how glia respond, and whether glial responses are involved in therapeutic effects are poorly understood. Here, we show that fluoxetine (FLX), an anti-depressant, mediates its anti-depressive effect by increasing the gliotransmission of ATP. FLX increased ATP exocytosis via vesicular nucleotide transporter (VNUT). FLX-induced anti-depressive behavior was decreased in astrocyte-selective VNUT-knockout mice or when VNUT was deleted in mice, but it was increased when astrocyte-selective VNUT was overexpressed in mice. This suggests that VNUT-dependent astrocytic ATP exocytosis has a critical role in the therapeutic effect of FLX. Released ATP and its metabolite adenosine act on P2Y_11_ and adenosine A2b receptors expressed by astrocytes, causing an increase in brain-derived neurotrophic factor in astrocytes. These findings suggest that in addition to neurons, FLX acts on astrocytes and mediates its therapeutic effects by increasing ATP gliotransmission.

## Highlights

• Anti-depressant FLX acts on astrocytes and increases VNUT-dependent ATP exocytosis.
• Such astrocytic responses are responsible for the FLX-induced therapeutic effects.
• Astrocytic ATP and its metabolite adenosine increase BDNF in astrocytes, and reveal the therapeutic effects.

Kinoshita et al. demonstrated that astrocytes are a therapeutic target of the antidepressant, fluoxetine (FLX). They found that FLX stimulates VNUT-dependent ATP release from astrocytes leading to a BDNF-mediated anti-depressive effect. This study demonstrated the astrocytic regulation of this anti-depressive effect, which complements the previously described conventional mechanism of FLX. Because the involvement of astrocytes in the pathogenesis of depression is of current interest, this new insight into the role of astrocytes in anti-depressive effects should support the establishment of novel therapeutic strategies for depression.

## 1 Introduction

Depression is a major public health problem worldwide. About 350 million people suffer from the disease and it will be ranked the second leading cause of death by the year 2020 [49]. There are several effective treatments for depression, but it is estimated that one-third of depressed patients do not respond adequately to conventional antidepressant drugs. Moreover, the slow onset of their therapeutic effects also restricts antidepressant use. Thus, there is an urgent need to identify the biological mechanism of depression and the pharmacological action of antidepressants. It is thought that antidepressants mediate their therapeutic effects by acting on neurons especially monoaminergic neurons, but they also act on non-neuronal cells such as glial cells. However, to date, how glial cells respond to antidepressants or whether glial responses are involved in the therapeutic effects of antidepressants remains unknown.

Astrocytes are the most abundant glial cells in the brain. In addition to their classical roles such as providing physical support to neurons or the removal of neuronal waste, astrocytes are active regulators of brain functions by releasing so-called “gliotransmitters” such as ATP, glutamate and d-serine [26]. Of these, ATP has received increased attention because it is released from astrocytes [25] and mediates various functions to regulate adjacent cells. In addition, released ATP is metabolized into adenosine, and both ATP and adenosine provide autocrine and paracrine signals via P2 and P1 receptors, respectively. Regarding the release of ATP, multiple pathways were reported, including connexin hemi-channels [16], pannexin hemi-channels [61], maxi-anion channels [40], P2X_7_ receptors [60] and exocytosis. Recently, Sawada et al. [51] reported vesicular nucleotide transporter (VNUT) uptakes ATP into intracellular vesicles. ATP was released by VNUT-dependent exocytosis in several types of cells including neurons [41], keratinocytes [30], microglia [29] and astrocytes [24, 35]. Astrocytic ATP has gained increasing attention because a recent report by Cao et al. clearly showed that decreased extracellular ATP mediated by astrocytes in the hippocampus caused depression in mice [6]. However, the mechanisms underlying the contribution of decreased ATP to depressive behavior, and whether anti-depressants affect astrocytic ATP functions, are poorly understood.

Brain-derived neurotrophic factor (BDNF) is increased by antidepressants and is considered to have a major role in the therapeutic action of antidepressants. For example, reduced BDNF levels were reported in depressed patients and models of depression, and antidepressant treatment increased BDNF expression [21]. It is well known that the majority of BDNF is produced by neurons [42] as well as microglial cells [28]. BDNF levels are very low in astrocytes from normal healthy adult brains, although other neurotrophic factors (glial cell–derived neurotrophic factor (GDNF) and nerve growth factor (NGF)) are synthesized in astrocytes [50]. However, BDNF was increased in response to changes in brain environment such as increased ATP [62], suggesting that astrocytes might be a source of BDNF during certain circumstances.

In the present study, we demonstrated that an antidepressant, fluoxetine (FLX), acts on astrocytes to mediate anti-depressive effects in mice. We also show that FLX increases extracellular ATP via VNUT, which subsequently increases BDNF in astrocytes. Thus, astrocytes and its related molecules depression are of great interest to understand further the therapeutic effects of FLX.

## 2 Materials and Methods

### 2.1 Animals

All experiments were carried out in accordance with the “Guiding Principles in the Care and Use of Animals in the Field of Physiologic Sciences” published by the Physiologic Society of Japan (LI, 2002) and with the approval of the Animal Care Committee of Yamanashi University (Chuo, Yamanashi, Japan). C57BL/6J mice (17-day-old fetuses or 9 week-old males) and Wistar rats (17-day-old fetuses) were obtained from Japan SLC (Shizuoka, Japan).

### 2.2 Generation of *Mlc1*-tTS BAC Transgenic Mice

The codons of bacterial tetracycline activator protein and human zinc finger protein KRAB domain were fully mammalianized (tTS). Mouse BAC DNA (clone RP23-114I6) was initially modified by inserting a Rpsl-Zeo cassette (gift from Dr. Hisashi Mori) into the translation initiation site of the *Mlc1* gene followed by replacement with a cassette containing tTS and SV40 polyadenylation signal. BAC DNA was linearized by PI-*Sce*I (Cat. # R0696S, New England Biolabs Inc., Massachusetts, U.S.A) enzyme digestion, and injected into fertilized eggs from CBA/C57BL6 mice.

### 2.3 Generation of VNUT-tetO Knock-in Mice

tetO responsive transgenes were constructed by placing a tetO responsive promoter element by use of 129 SvEv ES cells (Cat. # CMTI-1, RRID:CVCL_GS41). The tetO sequence was inserted upstream of the translation initiation site, and tetO insertion did not alter wild-type expression patterns [64]. Therefore, VNUT protein levels in VNUT-tetO homozygous mice were equivalent to those in wild-type mice.

### 2.4 Doxycyline-Mediated Control of Gene Expression in Double Transgenic Mice

We did not administer doxycycline to inhibit tTA- or tTS-mediated transcriptional control.

### 2.5 Generation of Astro-VNUT-KO and Astro-VNUT-OE Mice

We crossed *Mlc1*-tTS or *Mlc1*-tTA BAC transgenic mice with VNUT-tetO knock-in mice to generate Mlc-tTS::VNUT-tetO homozygous mice (astro-VNUT-KO), Mlc-tTA::VNUT-tetO homozygous mice (astro-VNUT-OE) and VNUT-tetO homozygous mice as littermate controls.

All mice were housed in plastic cages in groups of one to five per cage, at room temperature, and with free access to water and food. They were kept on an artificial 12 h light/dark cycle.

### 2.6 Experimental Schedule for Drug Treatment of Mice

FLX was freshly dissolved in saline before use. Animals were administered with FLX orally at a dose of 10 or 20 mg/kg or saline, using a volume of 10 ml/kg once daily for 21–28 days.

### 2.7 Measurement of Extracellular ATP in the Hippocampus

A previously described procedure for tissue ATP measurement [6] was used with some modifications. Briefly, mice were deeply anesthetized with pentobarbital and the hippocampal tissues were removed immediately. Transverse slices (300 μm thick) from the hippocampus were prepared using a tissue slicer (D.S.K. LINEARSLICER PRO7). Slices were immersed for 18 min in bubbled artificial cerebrospinal fluid (ACSF) composed of 125 mM NaCl, 5.0 mM KCl, 2.0 mM CaCl_2_, 2.0 mM MgSO_4_, 10 mM 2-[4-(2-Hydroxyethyl)-1-piperazinyl]ethanesulfonic acid (HEPES), 10 mM d-glucose and the ectonuclease inhibitor ARL67156 (100 mM) (95% oxygen and 5% carbon dioxide; 4 °C). Then the ACSF was collected and ATP levels were measured using an ATP determination kit (ATP Bioluminescence Assay Kit CLS II; Cat. # 11699695001, Roche Applied Science, Basel, Switzerland). Luminescence was measured by a luminometer (Berthold Lumat LB 9501). For normalization, protein amounts of each sample were measured by the bicinchoninic acid assay (Thermo Fisher Scientific, USA).

### 2.8 Tail Suspension Test

Animals were tested using a modified version of the tail suspension test (TST) that has been previously validated [58]. On the testing day, mice were brought into the behavior room 1 h before the test session to allow them to habituate to the environment. All experimental testing sessions were conducted between 12:00 P.M. and 6:00 P.M., with animals assigned and tested randomly. Eight FLX-treated animals were used, with a matched number of saline-treated control subjects. Each behavioral test was conducted 1 h after the previous drug injection. Mice were individually suspended by the tail with clamp (1 cm distant from the end) for 6 min in a box (MSC2007, YTS, Yamashita Giken, Tokushima, Japan) with the head 10 cm above the bottom of the box. Testing was carried out in a darkened room with minimal background noise. The duration of immobility was scored manually during a 6 min test. The behavioral measure scored was the duration of “immobility”, defined as the time when the mouse did not show any movement of the body and hanged passively.

### 2.9 Immunohistochemistry

After perfusion, brain segments were postfixed in 4% paraformaldehyde for 24 h, and then permeated with 20% sucrose in 0.1 M phosphate-buffered saline (PBS) (pH 7.4) for 24 h and 30% sucrose in 0.1 M PBS for 48 h at 4 °C. Brain segments were frozen in an embedding compound (Sakura Finetek, Tokyo, Japan) on dry ice. They were cut with a cryostat (Leica CM 1100; Leica, Wetzlar, Germany) at a thickness of 30 μm and collected in PBS at 4 °C to be processed immunohistochemically as free-floating sections. The sections were incubated for 48 h at 4 °C with primary antibodies: mouse anti-GFAP (1:2000; Cat. # AB5804, RRID: AB_10062746), rabbit anti-BDNF (1:2000; Cat. # sc-546, RRID:AB_630940). The sections were washed six times with 0.1 M PBS (10 min each) and then incubated for 3 h at room temperature with the secondary antibody: Alexa488- and Alexa546-conjugated mouse- and rabbit-IgGs.

(Cat. # A-11034, RRID:AB_2576217 and Cat. # A11030, RRID:AB_144695). Immunohistochemical images were obtained using a confocal laser microscope (Fluoview1000; Olympus, Tokyo, Japan) and digital images were captured with Fluoview1000 (Olympus).

### 2.10 Primary Cultures of Rat or Mouse Hippocampal Astrocytes

**Fig. 1:**
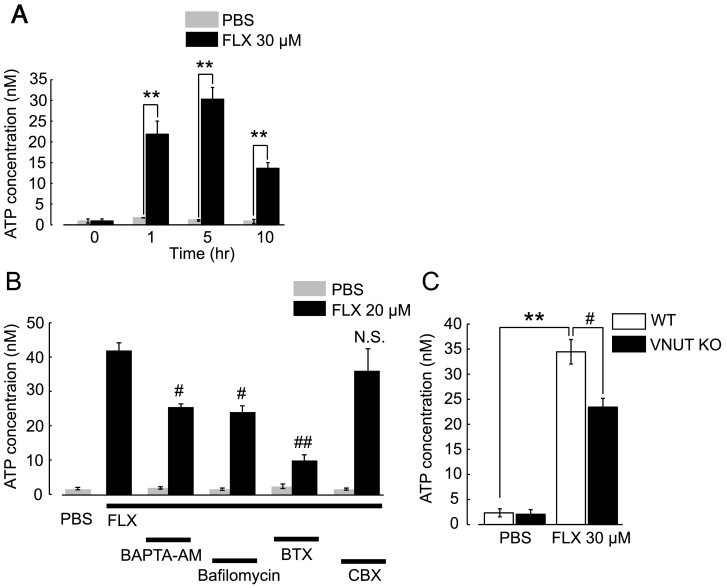
FLX-evoked increase in extracellular ATP via VNUT-dependent exocytosis in cultured hippocampal astrocytes. (A) The time-course of the FLX-evoked increase in extracellular ATP in hippocampal astrocytes in culture. Astrocytes were stimulated with FLX (30 μM) for 0, 1, 5 or 10 h at 37 °C, supernatants were collected, and then each ATP concentration was analyzed by luciferin-luciferase assay. Gray and black columns indicate PBS-treated control and FLX-treated astrocytes, respectively. **p < .01 vs. controls at each time point. (B) Pharmacological characterization of FLX-evoked ATP release. Astrocytes were treated with various inhibitors of exocytosis pathways (10 μM BAPTA-AM, 3 μM Bafilomycin) or connexin/pannexin hemi-channels (100 μM Carbenoxolone; CBX) for 30 min before and during FLX-stimulation in serum-free medium at 37 °C. Cells were pretreated with Botulinum toxin A (BTX, 5 U/ml) for 24 h. The concentration of ATP released from cultured astrocytes was measured 5 h after the application of FLX (20 μM). Gray and black columns indicate PBS-treated controls and FLX-treated astrocytes, respectively. ^#^*p* < .05, ^##^*p* < .01 vs. FLX alone. N.S.: not significant with *p* > .05 vs. FLX alone. (C) Effect of VNUT-deletion on FLX-evoked ATP release. Hippocampal astrocytes from WT and VNUT-KO mice were treated with FLX (30 μM) for 5 h, and then released ATP was measured. The FLX-evoked ATP increase in VNUT-KO astrocytes (black column) was significantly lower than in WT astrocytes (white column). Data are the mean ± SEM obtained from at least 3 independent experiments. **p < .01 vs. control (PBS), ^#^*p* < .05 vs. WT.

Primary cultures of astrocytes were derived from the hippocampus of newborn Wistar rats with the exception of Fig. 1C, which were from C57BL/6J mice and VNUT KO mice. Rat or mouse hippocampi were separated, minced, treated with 0.025% trypsin/EDTA (Gibco, NY) for 10 min at 37 °C, and then centrifuged for 10 min at 1000 ×*g*. The pellet was suspended in horse serum (Invitrogen, San Diego, CA), filtrated and cultured in 75 cm^2^ flasks in DMEM (Gibco, NY) containing 5% fetal bovine serum (Biological Industries, Kibbutz Beit-Haemek, Israel) and 5% horse serum at 37 °C in a 5% CO_2_ environment. After 10–13 days incubation, the culture was placed on a shaker and the cells were subjected to 24 h of continuous shaking to remove detached cells. Adherent astrocytes were detached by exposure to 0.1% trypsin/EDTA and then plated on 3.5-cm dishes and cultured in DMEM containing 5% fetal bovine serum and 5% horse serum at 37 °C in a 5% CO_2_ environment. Experiments were conducted with 5–7-day-old cultures.

### 2.11 Chemicals and Antibodies

Fluoxetine (FLX; Cat. # F132), imipramine (Cat. # I0899), mianserin (Cat. # M2525), paroxetine (Cat. # 1500218), adenosine 5′-triphosphate (ATP; Cat. # A26209), adenosine (Cat. # A9251), uridine 5′-triphosphate (UTP; Cat. # U6750), suramin (Cat. # S2671), reactive blue-2 (RB-2; Cat. # R115), pyridoxal phosphate-6-azobenzene-2,4-disulfonic acid (PPADS; Cat. # P178), MRS2179 (Cat. # M3808), α,β-methyleneadenosine 5′-triphosphate (α,βmeATP; Cat. # M6517), 2′,3′-O-(2,4,6-trinitrophenyl) adenosine 5′-triphosphate (TNP–ATP; Cat. # SML0740), 8-Cyclopentyl-1,3-dipropylxanthine (DPCPX; Cat. # C101), ARL67156 (Cat. # A265), H89 (Cat. # B1427), BAPTA-AM (Cat. # A1076), carbenoxolone (Cat. # C4790), bafilomycin A1 (Cat. # B1793) and norfluoxetine (NFLX; Cat. # F133) were from Sigma (St. Louis, Missouri, USA). KN93 (Cat. # 422711) and W7 (Cat. # 681629) were from Calbiochem (La Jolla, California, USA). SCH58261 (Cat. # 2270), MRS1706 (Cat. # 1584), MRS1220 (Cat. # 1217), NF340 (Cat. # 3830) and NF157 (Cat. # 2450) were from Tocris Bioscience (Ellisville, Missouri, USA). Botulinum toxin type A was from Allergan (Irvine, California, USA). All drugs were prepared as stock solutions in PBS or DMSO. The stocks were divided into single-use aliquots and stored at 4 °C or − 30 °C as required. In all experiments, the control groups without drugs received PBS or DMSO at a final concentration that matched the drug-containing solution. The maximum final DMSO concentration was 0.1%, and administering this concentration of DMSO had no effect on the expression of *Bdnf* mRNA compared with PBS alone (data not shown). NF340 stock solutions and Botulinum toxin type A were inactivated even at −30 °C. Therefore, solutions were made by dissolving in distilled water for each use, and used on the same day.

### 2.12 Quantitative PCR Analysis

Astrocytes were prepared in 35 mm dishes (4 × 10^5^ cells/dish) and total RNA was isolated and purified using NucleoSpin RNA II Kit (Cat. # U0955, Macherey-Nagel) according to the manufacturer's instructions. Reverse transcription (RT)-PCR was performed using a one-step PrimeScript RT-PCR Kit (Cat. # RR064, Takara Bio Inc., Shiga, Japan). The reaction mix contained 200 ng of total RNA, 200 nM primers, 100 nM TaqMan probe, TAKARA Ex Taq HS and PrimeScript RT enzyme mix. PCR assays were performed in 96-well plates on an Applied Biosystems 7500 (Applied Biosystems, Foster City, CA, USA). Reverse transcription was performed at 42 °C for 5 min followed by inactivation at 95 °C for 10 s. The temperature profile for PCR consisted of 40 cycles of denaturation at 95 °C for 5 s, and annealing/extension at 60 °C for 34 s. Primers and the TaqMan probes for rodent *Gapdh* (Cat. # 4308313) and *Bdnf* (Mm01334045_m1) were obtained from Applied Biosystems.

### 2.13 Luciferin-Luciferase ATP Assay

The bulk extracellular ATP concentration of astrocytes cultured in 24-well plates was measured by the luciferin-luciferase assay, as described previously (Wilharm et al., 2004), using an ATP Bioluminescence Assay Kit CLS II. This kit was used according to the manufacturer's recommendations. In brief, samples (100 μl for 24-well plates) were collected from each well at specified time points, boiled at 95 °C for 10 min, mixed with 100 μl of sample solution containing 100 μl of luciferin-luciferase reagent, and then photons were measured for 30 s by a luminometer at 20 °C. ATP standards provided with the kit were diluted in the range 10^−5^ to 10^−10^ M ATP. The no cells blank was subtracted from the raw data to calculate ATP concentrations from a log-log plot of the standard curve data.

### 2.14 Western Blotting

Astrocytes were lysed in lysis buffer (20 mM Tris-HCl pH 7.5, 2 mM EDTA, 0.5 mM EGTA, protease cocktail (Calbiochem, California, USA), 0.32 M sucrose). Cell lysates were resolved by SDS-PAGE and transferred to a nitrocellulose membrane (Bio-Rad, Tokyo, Japan). The membrane was blocked with 0.05% TBS-Tween and 5% skimmed milk (Wako Pure Chemical, Osaka, Japan) for 1–2 h at room temperature. Then, the membrane was probed with rabbit anti-BDNF antibody (Cat. # sc-20,981, RRID:AB_2064213) diluted at 1:4000 in can get signal solution (Cat. # NKB-101, Toyobo, Osaka, Japan), rabbit anti-CREB (Cat. # 9197, RRID:AB_331277) or phospho-CREB (Cat. # 9198, RRID:AB_2561044) diluted at 1:4000 in can get signal solution, or mouse anti-rat β-actin (Cat. # A5316, RRID:AB_476743) diluted at 1:15,000 in can get signal solution. Anti-BDNF, CREB or phospho-CREB primary antibodies were detected using horseradish peroxidase-conjugated anti-rabbit IgG (Cat. # NA934; RRID:AB_772206), diluted at 1:1000 in can get signal solution, and anti-rat β-actin antibodies were detected using horseradish peroxidase-conjugated anti-mouse IgG (Cat. # NA931; RRID:AB_772210) diluted at 1:10,000 or 1:30,000 in can get signal solution. Images were visualized with an ECL system (GE Healthcare Biosciences) or Super Signal West Femto Maximum Sensitivity Substrate (Cat. # 34095, Thermo Scientific).

### 2.15 Primary Culture of Rat Hippocampal Neurons

Primary cultures of neurons were derived from the hippocampus of newborn Wistar rats. Rat hippocampi were separated, minced, and digested in Neuron Dissociation Solutions Kit (Cat. # 291–78,001, Wako Pure Chemical) according to the manufacturer's protocol. Neurons were dispersed in DMEM containing 5% fetal bovine serum and 5% horse serum and maintained under an atmosphere of 10% CO_2_ at 37 °C. The culture medium was changed twice a week and neurons were used 14 days after plating.

### 2.16 Purification of Astrocytes by Magnetic-Activated Cell Sorting (MACS)

Purification of astrocytes from adult mouse brain was performed with MACS technology using an adult brain dissociation kit (130-107-677, Miltenyi Biotec, Bergisch Gladbach, Germany) and a MCASmix™ Tube Rotator (130–090-753) following the manufacturer's protocol. Mice were anesthetized with 50 mg/kg pentobarbital (i.p. injection) and transcardially perfused with ice-cold 0.1 M PBS. The brain was chopped into small pieces (approximately 1 mm) with surgical scissors and digested in 1900 μl of buffer Z containing buffer Y (20 μl), enzyme A (10 μl) and P (50 μl) using the dissociation program of the tube rotator. Then 20 ml of ice-cold PBS containing 0.5% (wt/vol) BSA (PBS/BSA) was added, mixed, and samples were filtered through a cell strainer (100 μl). Samples were centrifuged at 300 ×*g* for 7 min at 4 °C and the supernatant was discarded. The pellet was resuspended in 3100 μl of PBS/BSA and 900 μl of debris removal solution (130–109-398) was added followed by 4 ml PBS/BSA and centrifugation at 3000 ×*g* for 10 min at 4 °C. The supernatant was aspirated and 15 ml of PBS/BSA was added and the solution was mixed well. Samples were centrifuged at 300 ×*g* for 7 min at 4 °C and the supernatant was discarded. Then 80 μl of PBS/BSA and 10 μl of FcR blocking buffer were added followed by 10 μl of anti-astrocyte cell surface antigen-2 (ACSA-2) microbeads (130–097-678). Samples were incubated for 15 min at 4 °C, centrifuged at 300 ×*g* for 7 min at 4 °C, and the cells were resuspended in 500 μl PBS/BSA. The cells were then transferred to an LS column (130-042-401). The column was set on a magnetic stand and 3 ml PBS/BSA was added three times. The column was removed from the magnet and 3 ml PBS/BSA was added. The flow through was collected as the ACSA-2-negative (ACSA-2-) fraction. Another 3 ml of PBS/BSA was then added to the column and the fraction containing the anti-ACSA-2-attached astrocytes was collected. Using this technique, the ACSA-2-positive fraction had significantly higher *Gfap* mRNA levels (>30 fold) compared with the ACSA-2- fraction (Fig. S1B and S1C), indicating the successful purification of astrocytes from the adult mouse brain.

### 2.17 Statistics

Data were presented as the mean ± SEM, from *n* ≥ 3 independent determinations performed in duplicate. Significance of differences between data obtained for control samples and each sample treated with reagents was determined using ANOVA, followed by Tukey's test for multiple comparisons. Unpaired and paired *t*-tests were used for the comparison of two groups. Differences were considered significant when the *P* value was <0.05.

## 3 Results

### 3.1 FLX Stimulates the Exocytosis of ATP in Astrocytes

As reported by Cao et al., a decrease in extracellular ATP in the hippocampal astrocytes caused depression, which was restored by exogenously applied ATP [6]. To investigate whether the antidepressant FLX increases extracellular ATP in astrocytes, primary cultures of hippocampal astrocytes were stimulated with FLX. As shown in Fig. 1A, FLX increased extracellular ATP, which reached a maximal level (30.5 ± 2.8 nM) 5 h after FLX stimulation. Multiple pathways or mechanisms have been reported for ATP release in glial cells, such as maxi-anion channels [40], P2X_7_ receptors [60], connexin and pannexin hemi channels [16, 61] and exocytosis [44]. The FLX-evoked increase in extracellular ATP was significantly reduced by a Ca^2+^ chelator BAPTA-AM (10 μM), a V-ATPase inhibitor bafilomycin (3 μM) and a SNAREs inhibitor Botulinum toxin A (BTX, 5, 10 U/ml), but not by the connexin/pannexin inhibitor, carbenoxolone (CBX, 100 μM), suggesting the involvement of exocytosis (Fig. 1B). Astrocytes express soluble N-ethylmaleimide–sensitive factor attachment protein receptors (SNAREs) such as synaptobrevin, syntaxin I and SNAP-23 [67] and release ATP by an intracellular Ca^2+^ dependent mechanism [11]. In addition, Sawada et al. recently identified a vesicular nucleotide transporter (VNUT or *Slc17a9*), an essential molecule for vesicular storage and release of ATP [51]. FLX-evoked ATP release was significantly inhibited in astrocytes obtained from VNUT-knockout (VNUT-KO) mice (Fig. 1C, WT; 34.5 ± 2.4 nM vs VNUT-KO, 22.4 ± 2.3 nM) suggesting that FLX at least in part induces the release ATP by VNUT-dependent exocytosis. Although BTX almost abolished FLX-induced ATP release in astrocytes, BAPTA-AM, bafilomycin, and VNUT-KO astrocytes also partially reduced ATP release (by approximately 45%, 49%, and 34%, respectively). These findings suggest that mechanisms other than exocytosis are also involved in ATP release.

### 3.2 FLX Increased Extracellular ATP and Induced Anti-Depressive Behavior Via Astrocytic VNUT

**Fig. 2:**
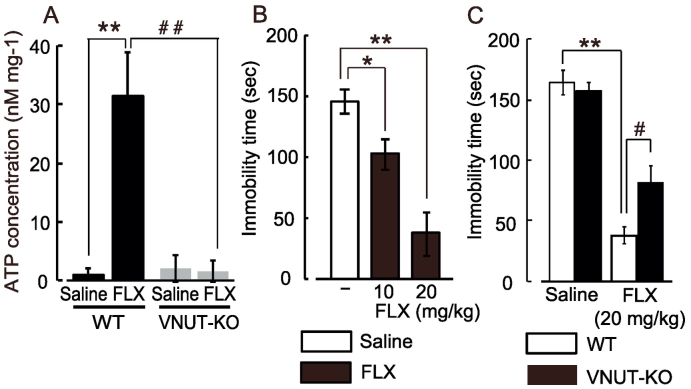
Extracellular ATP within the hippocampus and FLX-induced anti-depressive behavior caused by VNUT-deletion. (A) Measurement of extracellular ATP content in the artificial cerebrospinal fluid (ACSF) media of hippocampal slices taken from WT and VNUT-KO mice 21 days after the chronic administration of FLX (mean ± SEM, *n* = 4). **p < .01 vs. WT saline group, ^##^*p* < .01 vs. littermate control FLX group. (B) Anti-depressive effects in mice were assessed by TST. WT (C57BL/6 J) mice were administered with saline or FLX (10 and 20 mg/kg per day, p.o.) for 3 weeks. TST was performed 1 h after the final FLX-administration. Data show the mean ± SEM of immobility times (*n* = 6). *p < .05 and **p < .01 vs. saline. (C) VNUT-KO mice (C57BL/6 J background) were administered with saline or FLX (20 mg/kg per day) for 3 weeks, and TST was performed. Data show the immobility times (mean ± SEM, *n* = 5). ^#^*p* < .05 vs. WT, **p < .01 vs. saline group.

To determine whether FLX affects the amount of ATP in vivo, we measured the concentration of ATP in the ACSF from acute hippocampal slices of FLX-administered mice. The chronic administration of FLX markedly upregulated the amount of ATP in the hippocampus of WT mice, while this increase was completely blocked in VNUT-KO mice (Fig. 2A). These results indicate that increased ATP release by FLX is dependent on VNUT.

ATP derived from astrocytes modulated depressive behaviors in mice [6]. To elucidate whether astrocytic ATP gliotransmission facilitated by FLX mediates its therapeutic effects, we tested FLX-induced anti-depressive effects in VNUT-KO mice. FLX at 10 and 20 mg/kg was administered for 21 days in wildtype (WT) control mice, and its therapeutic effect was assessed by tail suspension test (TST). As shown in Fig. 2B, chronic administration of FLX (21 days) significantly decreased immobility time in a concentration-dependent manner over a concentration range from 10 to 20 mg/kg (Saline [control] 145.4 ± 11.6 s vs. FLX at 20 mg/kg 38.4 ± 18.4 s), indicating FLX induced anti-depressive effects in mice. These results correspond well with a previous report [14], and thus we chose FLX (20 mg/kg) administered for 21 days for the following experiments.

**Fig. 3:**
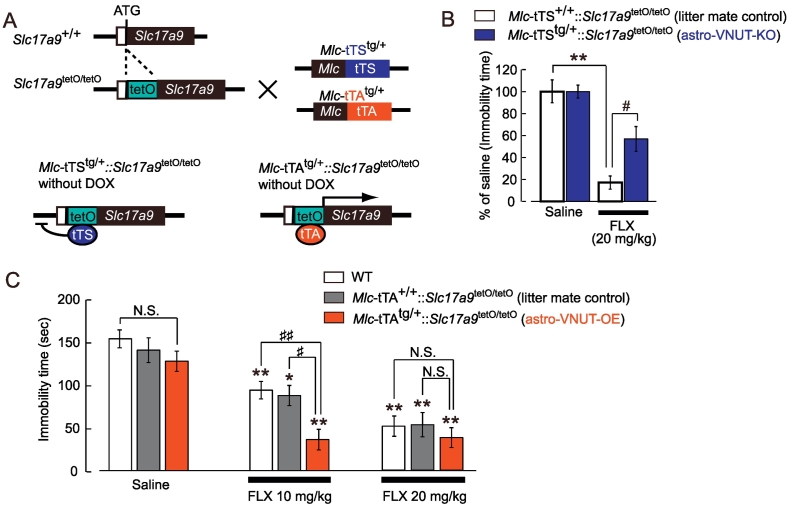
Astrocytic modulation of anti-depressive effects depends on VNUT expression. (A) Schematic diagram of astrocyte-specific tTS-mediated knockout (KO) and tTA-mediated overexpression (OE) systems. *Mlc1*-tTS; astrocyte-specific *Mlc1* promoter drives the expression of the tetracycline-controlled transcriptional silencer (tTS). *Mlc1*-tTA; *Mlc1* promoter drives the expression of the tetracycline-controlled transcriptional activator (tTA). TetO-*Slc17a9* contains a tet operator (tetO)–regulated VNUT gene, i.e., *Slc17a9*. By crossing tetO-*Slc17a9* with either *Mlc1*-tTS or *Mlc1*-tTA, we generated *Mlc*-tTS^tg/+^::*Slc17a9*^tetO/tetO^, an astrocyte-specific VNUT-KO mouse (astro-VNUT-KO), *Mlc*-tTA^tg/+^::*Slc17a9*^tetO/tetO^, an astrocyte-specific VNUT OE mouse (astro-VNUT-OE), and *Mlc*-tTA^+/+^::*Slc17a9*^tetO/tetO^, their littermate control mouse (littermate control) whose VNUT expression is the same as WT. (B and C) Anti-depressive effect of FLX in astro-VNUT-KO mice (B) and astro-VNUT-OE mice (C). Mice were administered saline or FLX (10 or 20 mg/kg, p.o.) for 3 weeks and TST was performed. Data show the mean ± SEM of immobility times obtained from at least 4 independent set of experiments. *p < .05, **p < .01 vs. saline-treated group, ^#^*p* < .05, ^##^*p* < .01 vs. littermate control, N.S., not significant.

When FLX (20 mg/kg) was administered for 21 days in VNUT-KO mice, its anti-depressive effect, as measured by a decrease in immobility time, was significantly weaker than in WT mice (Fig. 2C). When saline was administered, there was no significant difference in immobility time between WT and VNUT-KO mice. As shown in Fig. 1, FLX caused VNUT-dependent ATP exocytosis from hippocampal astrocytes; therefore, we generated double-transgenic mice from astrocyte-specific tetracycline trans-silencer (tTS) or tetracycline trans-activator (tTA) lines and VNUT tetO knockin lines for astrocyte-specific gene knockout or overexpression. Astrocytes purified from the adult brains of Mlc-tTA::VNUT-tetO or Mlc-tTS::VNUT-tetO mice using MACS exhibited significantly increased or reduced *Slc17a9* mRNA levels (1020-fold increase or 2.6-fold decrease, respectively) (Fig. S1), whereas no changes were detected in other cell types (Fig. S1D, E). Hereafter, we refer to Mlc-tTA::VNUT-tetO (astrocyte-selective VNUT-overexpression) and Mlc-tTS::VNUT-tetO (astrocyte-selective VNUT-KO mice) mice as astro-VNUT-OE and astro-VNUT-KO mice, respectively. We investigated the effect of astrocyte-selective VNUT-deletion on FLX-evoked anti-depressive effects using astro-VNUT-KO (Fig. 3A). There was no significant difference in immobility time between astro-VNUT-KO mice and their littermate control mice when treated with saline. However, similar to VNUT-KO, FLX-evoked anti-depressive effects were significantly weaker in astro-VNUT-KO mice than in littermate control mice (astro-VNUT-KO, 86.6 ± 5.1 vs. littermate control, 49.3 ± 9.8; immobility time, *sec*, *p < .05) (Fig. 3B). We tested the effect of astrocytic VNUT overexpression on the FLX-evoked anti-depressive effect using astro-VNUT-OE mice. At 20 mg/kg, the FLX-induced anti-depressive effect in astro-VNUT-OE mice was similar to that in WT mice and littermate controls (Fig. 3C). However, at 10 mg/kg, the FLX-induced anti-depressive effect was significantly stronger in astro-VNUT-OE mice than WT or littermate control mice (Fig. 3C, middle). Thus, a decrease and increase in VNUT in astrocytes correlated with the decrease and increase in the anti-depressive effects of FLX, respectively. There was no significant difference in basal immobility time when tested before and after saline administration among any mutant mice used in these studies (WT, litter mate control mice, astro-VNUT-KO mice and astro-VNUT-OE mice) (data not shown). These findings strongly suggest that FLX acts on astrocytes to control VNUT-dependent ATP exocytosis, which mediates its therapeutic effect, at least in part.

### 3.3 FLX and Other Antidepressants Induce BDNF in a Primary Culture of Hippocampal Astrocytes

**Fig. 4:**
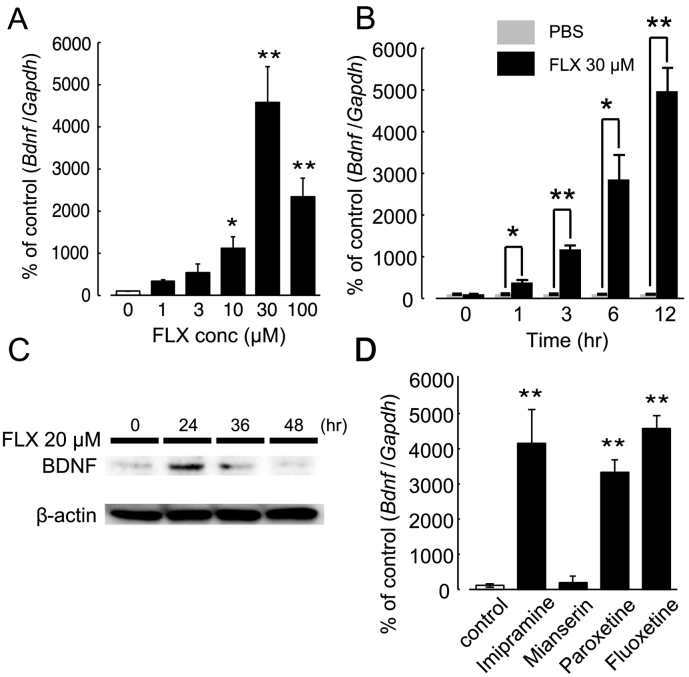
FLX-evoked increase of BDNF in astrocytes. (A) Dose-dependent enhancement of *Bdnf* mRNA in cultured hippocampal astrocytes by FLX. Cells were incubated with various concentrations of FLX (1–100 μM) or PBS for 12 h in serum-free culture medium. Maximal *Bdnf* mRNA expression was observed with 30 μM of FLX (*p < .05, **p < .01 vs. control (PBS)). Values are normalized to the PBS-treated control, and shown as a % of the control. Data show the mean ± SEM of representative triplicate measurements. Three independent experiments were performed and similar results were obtained. (B) Time-course of the FLX-evoked increase of *Bdnf* mRNA in astrocytes. Cells were incubated with FLX (30 μM) or PBS for different periods (from 0 to 12 h) in serum-free culture medium. Gray and black columns indicate the PBS- and FLX-treated groups, respectively. Values are normalized to the PBS-treated control and shown as a % of the control. Data show the mean ± SEM of a representative triplicate measurement. Two independent experiments were performed and similar results were obtained. *p < .05, **p < .01 vs. PBS-treated group. (C) Western blot analysis of FLX-evoked BDNF in hippocampal astrocytes. Cells were treated with FLX (20 μM) for 24, 36 or 48 h, and protein levels of BDNF were assessed by western blot (WB) analysis. Data show a representative WB band of BDNF (upper) and β-actin (lower). At least 3 independent experiments were performed and similar results were obtained. (D) Effect of other anti-depressants on the expression of *Bdnf* mRNA in hippocampal astrocytes. Cells were treated with different anti-depressants including Imipramine 30 μM, Mianserin 30 μM, Paroxetine 30 μM and Fluoxetine 30 μM for 12 h. *Bdnf* mRNA levels in each group were measured and compared. Data show the mean ± SEM of the % of controls (without anti-depressants), showing representative triplicate measurements.

To address the mechanisms underlying the astrocytic VNUT-mediated anti-depressive effect of FLX, we focused on extracellular ATP and BDNF because FLX increased ATP in astrocytes (Fig. 1) and ATP increased the astrocytic expression of *Bdnf* mRNA [62], one of the most important molecules in the pathogenesis of depression [72]. Fig. 4A and B show the dose- and time-dependency of the FLX-evoked increase in *Bdnf* mRNA in a primary culture of hippocampal astrocytes. Treatment with FLX for 6 h increased *Bdnf* mRNA in a concentration-dependent manner (1–30 μM) (Fig. 4A), and at 12 h after 30 μM FLX administration, it reached 4270% of the PBS-treated control (*p* < .01). The FLX-evoked increase in *Bdnf* mRNA was initiated at 1 h and gradually increased to at least 12 h after FLX administration (Fig. 4B). FLX also increased BDNF protein levels, which reached a maximal level 24 h after FLX treatment (Fig. 4C). We investigated the effects of other antidepressants on *Bdnf* mRNA expression in cultured hippocampus astrocytes. As shown in Fig. 4D, treatment with imipramine (30 μM), paroxetine (30 μM) and FLX (30 μM) for 12 h significantly increased the expression of *Bdnf* mRNA, but mianserin (30 μM) did not. Imipramine is a tricyclic antidepressant, paroxetine and FLX are classified as selective serotonin reuptake inhibitors (SSRIs), and mianserin is a tetracyclic antidepressant. These results suggest that increased BDNF in astrocytes might be a common pharmacological feature across different types of antidepressants.

FLX is a pro-drug, and is metabolized into norfluoxetine (NFLX), which then mediates its pharmacological effects [57]. Therefore, we tested the effect of NFLX on *Bdnf* mRNA expression in hippocampal astrocytes. NFLX increased *Bdnf* mRNA in a concentration-dependent manner (10 and 30 μM) (Fig. S2A).

In cell cultures, the concentration of FLX and other antidepressants used (20–30 μM) mostly exceeded the therapeutic plasma levels in patients (1–3 μM), indicating the effects of antidepressants in this study might be overestimated. However, concentrations of FLX in the human brain were reported to be 20-fold higher than those in the plasma [34], indicating that an FLX concentration of 30 μM might occur in the brain.

### 3.4 Chronic Administration of FLX Increases BDNF in Hippocampal Astrocytes In Vivo

**Fig. 5:**
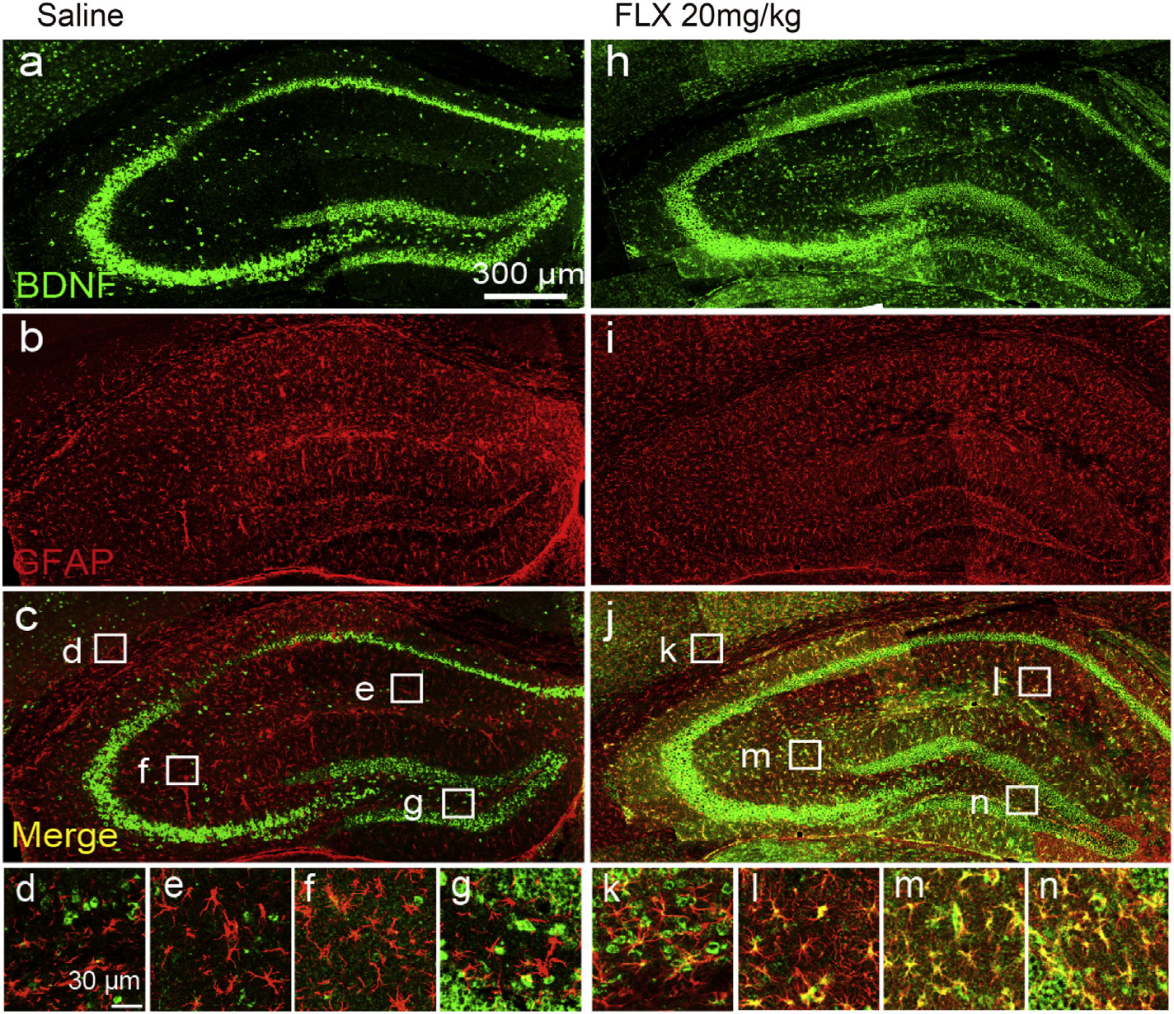
Chronic administration of FLX upregulates BDNF in hippocampal astrocytes. Double immunostaining of hippocampal sections with anti-BDNF (green) and anti-GFAP (red) antibodies. *a-c* are images of saline administered mice. *h-j* are FLX (20 mg/kg for 21 days, p.o.) administered mice. *d-g* and *k-n* are the magnified insets shown in *c* and *j*, respectively. Scale bars: *a,* 300 μM; *d*, 30 μM. These are representative of at least 3 independent experiments where similar results were obtained.

To determine whether FLX increases BDNF in astrocytes in vivo, we measured BDNF expression in astrocytes by immunohistochemical analysis. After chronic administration of FLX (20 mg/kg for 21 days), brain sections were stained with anti-BDNF and anti-GFAP (glial fibrillary acidic protein) antibodies (Fig. 5). In saline-administered mice, BDNF-immunoreactivities were predominantly observed in neurons of the granule cell layer, dentate gyrus and pyramidal cell layers in CA1, CA2 and CA3 (Fig. 5a) but little BDNF-immunoreactivity was observed in GFAP-positive astrocytes (Figs. 5d-g). After chronic FLX-administration, however, BDNF-positive signals were increased in neurons, and in astrocytes across all regions of the hippocampus (Figs. 5h-n), indicating FLX increased BDNF in astrocytes in vivo. In comparison to the hippocampus, astrocytes showed only a slight increase in BDNF-immunoreactivity in the cortex (not shown) indicating that the FLX-induced BDNF increase in astrocytes is partly dependent on brain region. This regional difference in BDNF expression was also observed in vitro, where FLX-induced *Bdnf* mRNA upregulation was significantly higher in hippocampal astrocytes than in cortical astrocytes (Fig. S3).

We measured the BDNF expression in VNUT-KO mice (Fig. S4). In contrast to WT mice, BDNF-positive signals were less elevated in GFAP-positive astrocytes despite the chronic administration of FLX, indicating that FLX-induced astrocytic BDNF expression in vivo depends on VNUT. We also examined whether microglia express BDNF by immunohistochemistry after the chronic administration of FLX. CD11b positive microglia did not express BDNF and CD11b staining did not show any morphological changes such as the retraction of processes or hypertrophic cell bodies and processes, which are characteristic of activated microglia (data not shown). These data indicate that the major sources of BDNF in our model are astrocytes and neurons, but not microglia.

### 3.5 FLX-Evoked BDNF Upregulation in Astrocytes Is Mediated by Activation of P2 and P1 Receptors

**Fig. 6:**
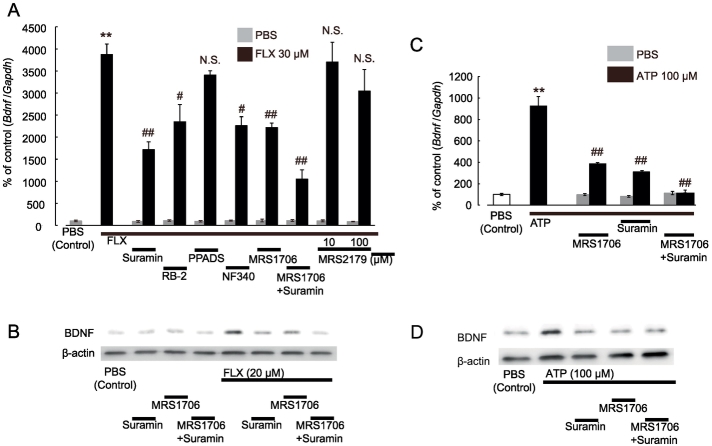
Involvement of purinergic receptors in the FLX-evoked increase of BDNF in hippocampal astrocytes. Hippocampal astrocytes were treated with several purinergic receptor antagonists (100 μM suramin, 10 μM RB-2, 100 μM PPADS, 30 μM NF340, and 1 μM MRS1706) for 30 min before and during FLX (12h) or ATP (1 h) stimulation. (A) Effects of various antagonists on the FLX (30 μM)-evoked increase of *Bdnf* mRNA in astrocytes. Cells were treated with FLX for 12 h. Gray and black columns indicate the PBS-treated control and FLX-treated groups, respectively. **p < .01 vs. PBS-treated control. ^#^*p* < .05, ^##^*p* < .01 vs. FLX alone. N.S., not significant, *p* > .05 vs. FLX alone. Values are normalized to PBS-treated controls, and are shown as a % of the control. Data show the mean ± SEM of 3 independent experiments. (B) Western blot analysis of the FLX-evoked increase of BDNF protein in astrocytes. Astrocytes were treated with FLX (20 μM) for 24 h with and without various antagonists. The WB bands show representative data from 3 independent experiments. (C) Effects of various antagonists on the ATP (100 μM)-evoked increase of *Bdnf* mRNA in astrocytes. Cells were treated with ATP for 1 h. Gray and black columns indicate the PBS-treated control and ATP-treated groups, respectively. **p < .01 vs. PBS-treated control. ^#^*p* < .05, ^##^*p* < .01 vs. ATP alone. N.S., not significant, *p* > .05 vs. ATP alone. Values are normalized to PBS-treated controls, and shown as a % of the control. Data show the mean ± SEM of 3 independent experiments. (D) Western blot analysis of the ATP-evoked increase in BDNF protein in hippocampal astrocytes. Astrocytes were incubated with ATP (100 μM) for 6 h with and without various antagonists. The WB bands show representative data from 3 independent experiments.

Next, we investigated the mechanisms underlying the FLX-evoked increase in BDNF in astrocytes, with a focus on extracellular ATP-mediated signals, because FLX increases extracellular ATP. The FLX-evoked increase in *Bdnf* mRNA in astrocytes was significantly decreased by the non-selective P2 receptor antagonist suramin or RB-2, a P2Y_11_ receptor antagonist NF340, but not by a P2X receptor antagonist pyridoxal phosphate-6-azobenzene-2,4-disulfonic acid (PPADS) or a P2Y_1_ receptor antagonist MRS2179. In addition, the upregulation of *Bdnf* mRNA was also inhibited by an adenosine A2b receptor antagonist MRS1706, which was further inhibited when simultaneously applied with suramin (Fig. 6A). This suggested that both P2 and P1 receptors, especially P2Y_11_ and A2b receptors, are involved in BDNF production. Similar results were obtained from the western blot analysis of BDNF (Fig. 6B). To confirm the involvement of ATP in BDNF upregulation, hippocampal astrocytes were directly stimulated with ATP. We observed increased *Bdnf* mRNA and BDNF proteins in astrocytes, which was decreased by treatment with MRS1706 or suramin alone, or their co-application which further decreased BDNF (Fig. 6C, D). In addition, the NFLX-evoked increase in *Bdnf* mRNA was also inhibited by suramin or MRS1706 (Fig. S2A).

P2 receptors are classified into ion channel forming P2X receptors and G-protein coupled P2Y receptors, which are subdivided into P2X_1–7_ and P2Y_1,2,4,6,11,12,13,14_ receptors [5]. Thus, we further performed pharmacological analysis (Fig. S5). Trang et al. [65] reported that BDNF was upregulated by the activation of P2X_4_ receptors in microglia. However, an agonist to P2X receptors, α,β methyleneATP (α,βmeATP) [23] failed to upregulate BDNF (Fig. S5D), and the FLX-evoked BDNF increase in astrocytes was not reduced by a P2X_1–4_ receptor antagonist, TNP-ATP and P2X_4_ receptor-selective antagonist, 5-BDBD (Fig. S5B). These findings indicate the involvement of P2X_4_ receptors in BDNF production in astrocytes is negligible. Based on our pharmacological analyses (Fig. S5), we concluded that the P2Y_11_ receptor is the most probable P2 receptor involved in BDNF induction in hippocampal astrocytes. However, suramin (100 μM) and RB-2 (10 μM) reduced the ATP-evoked BDNF induction by 72.3 ± 1.5% (*p* < .01) and 65.4 ± 0.5% (*p* < .01), respectively, whereas NF340 or NF157 had a reduced inhibitory effect (44.3 ± 1.9% inhibition, *p* < .05 and 46.6 ± 2.0% inhibition, *p* < .05 inhibition, respectively). This may also suggest the existence of additional P2 receptors involved in BDNF upregulation.

Regarding P1 receptors, we also performed additional pharmacological analyses. Extracellular ATP is rapidly metabolized into ADP, AMP and adenosine by NTPDases and 5′-nucleotidase [70]. Unlike ATP or ADP, adenosine acts on P1 receptors such as A1, A2a, A2b, and A3 adenosine receptors. When hippocampal astrocytes were treated with adenosine directly, *Bdnf* mRNA was increased in a dose-dependent manner over a concentration range from 1 to 100 μM. The ED50 value was approximately 4.1 μM (Fig. S6A), suggesting the involvement of a low-affinity adenosine receptor subtype, possibly the A2b receptor [45]. Adenosine also increased BDNF protein levels in astrocytes (Fig. S6D). The time-course of adenosine-evoked *Bdnf* mRNA upregulation was transient and peaked at 1 h after stimulation (Fig. S6B). This time course was similar to that of ATP (Fig. S5A) and faster than that of FLX (Fig. 4B). Adenosine-evoked increases in *Bdnf* mRNA in astrocytes were inhibited by an A2b receptor antagonist, MRS1706, but not by A1 (DPCPX), A2a (SCH58261), or A3 (MRS1220) receptor antagonists (Fig. S6C). All these pharmacological profiles strongly suggest that A2b receptors are responsible for BDNF induction.

FLX and other SSRI antidepressants inhibit the uptake of serotonin (5-HT), and increase 5-HT-mediated neuronal responses involving BDNF upregulation in neurons [20]. In our study using a neuronal culture, FLX upregulated *Bdnf* mRNA (632.6 ± 17.1% vs. PBS-treated control), while suramin and MRS1706 did not suppress its increase (754.9 ± 63.2% and 725.1 ± 64.3% vs. PBS-treated control, respectively) (Fig. S7). As a control experiment, we stimulated neurons with 1 or 10 μM 5-HT, and found that 5-HT elicited *Bdnf* mRNA expression (1 μM: 234.3 ± 49.6% and 10 μM: 449.1 ± 131.5% vs. PBS-treated control). These data demonstrate that FLX also increases BDNF in neurons, which is not dependent on purinergic receptors, but is probably dependent on 5-HT signals via the inhibition of 5-HT uptake.

Next, we investigated the effect of 5-HT on BDNF expression in astrocytes. When treated with 5-HT (0.1, 1, 10 μM) for 1 and 6 h, astrocytes did not upregulate *Bdnf* mRNA (Fig. S5E) indicating no involvement of 5-HT in astrocytic BDNF production. Thus, the FLX-induced BDNF increase in astrocytes appears to be dependent on extracellular ATP and adenosine, and activation of their corresponding receptors, P2Y_11_ and A2b, respectively.

### 3.6 FLX-Evoked BDNF Upregulation Is Mediated by cAMP/PKA in Hippocampal Astrocytes

**Fig. 7:**
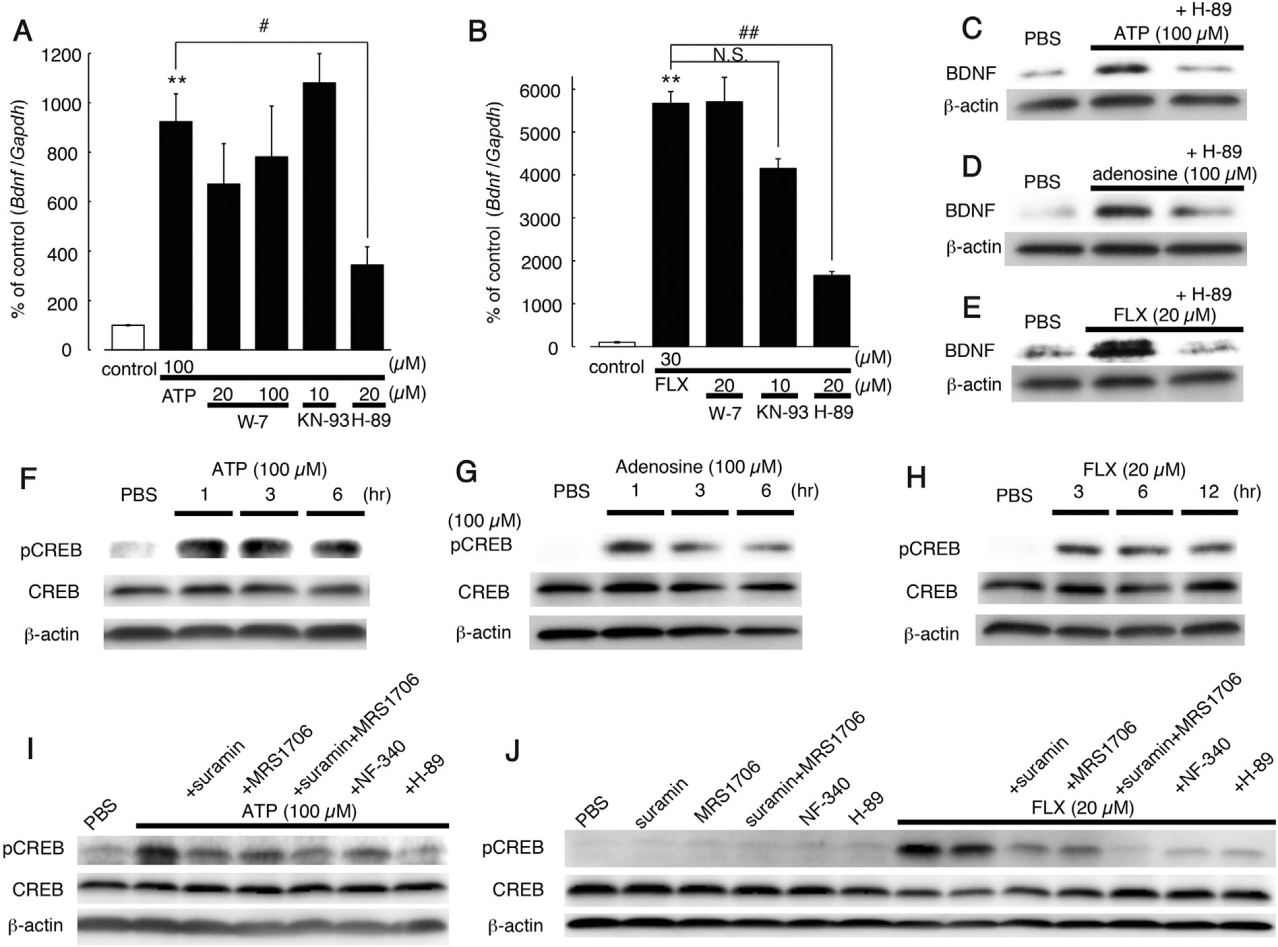
Involvement of the PKA-pCREB pathways in FLX- and ATP-evoked BDNF production in astrocytes. (A and B) Effects of various kinase inhibitors on the ATP (A)- and FLX (B)-evoked increase of *Bdnf* mRNA in hippocampal astrocytes. Cells were treated with a PKA inhibitor H-89 (20 μM), a CaMKII inhibitor KN-93 (10 μM) or a calmodulin inhibitor W-7 (20 or 100 μM) for 30 min before and during ATP (100 μM for 1 h) or FLX (20 μM for 12 h) stimulation. Values were normalized to the PBS-treated controls, and shown as a % of the control. Data show the mean ± SEM. **p < .01 vs. PBS-treated control. ^#^*p* < .05 vs. ATP-treated. ^##^*p* < .01 vs. FLX-treated, N.S., not significant, *p* > .05 vs. FLX-treated. (C − E) Western blot analysis, showing the effect of H-89 on the ATP- (C), adenosine- (D) and FLX- (E) evoked increase of BDNF in hippocampal astrocytes. H-89 (20 μM) was added to cells for 30 min before and during ATP (6 h), adenosine (6 h) and FLX (24 h) stimulation. Data show representative western blotting data obtained from 3 independent experiments. (F − H) Western blot analysis of the phosphorylation of CREB (pCREB) by ATP (F), adenosine (G) and FLX (H) in astrocytes. Hippocampal astrocytes were treated with 100 μM ATP (F), 100 μM adenosine (G) and 20 μM FLX (H) for the times indicated (1−12h). Changes in pCREB, CREB, and β-actin proteins in response to these stimuli are shown. (I and J) Western blot analysis showing the effects of suramin, MRS1706, NF-340 and H-89 on ATP (I)- or FLX (J)-evoked pCREB formation in hippocampal astrocytes. Suramin (100 μM), MRS1706 (1 μM), NF-340 (10 μM) and H-89 (20 μM) were incubated with cells for 30 min before and during 100 μM ATP (1 h) or 20 μM FLX (6 h) stimulation. Changes in pCREB, CREB, and β-actin protein are shown. These are representative western blot data obtained from at least 3 independent experiments.

We further investigated the intracellular signaling cascades of the FLX-evoked BDNF increase in astrocytes. We showed that both P2Y_11_ and A2b receptors are involved in the FLX-evoked responses. These receptors are coupled with Gs proteins, the activation of which results in the accumulation of cAMP and activation of protein kinase A (PKA) [12, 13, 22]. In addition, both receptors also mobilize intracellular calcium and activate Ca^2+^/calmodulin-dependent kinase (CaM kinase) [63]. P2Y_11_ receptors are coupled to Gs proteins as well as Gq proteins, leading to the mobilization of Ca^2+^ from inositol 1,4,5-trisphosphate [Ins(1,4,5)P3]-sensitive stores [68]. A2b receptors evoked a phospholipase C-dependent increase in intracellular Ca^2+^ [47] by Gq-dependent or -independent mechanisms [22]. However, a CaM kinase inhibitor, KN-93, and a calmodulin antagonist, W-7, did not inhibit the FLX- or ATP-evoked upregulation of *Bdnf* mRNA in astrocytes. However, a PKA inhibitor H-89 [10] significantly reduced ATP- and FLX-evoked responses by 62.8 ± 7.9% and 70.8 ± 1.6%, respectively, suggesting the involvement of PKA-mediated intercellular mechanisms in FLX- and ATP-induced BDNF expression (Fig. 7A, B).

It is well known that *Bdnf* transcription is controlled by cAMP response element-binding transcription factor (CREB), which is phosphorylated by PKA (Carlezon et al., [7]). Indeed, phosphorylated CREB (pCREB) promoted BDNF gene transcription [18], and the cAMP-PKA-CREB pathway stimulated the transcription of *Bdnf* in astrocytes [8, 32, 71]. Stimulation of either P2Y_11_ receptors [66] or A2b receptors [37, 52] activated cAMP-CREB pathways in several cell types. Both ATP and adenosine increased pCREB in hippocampal astrocytes with a peak expression at 1 h (Figs. 7F, G). FLX also increased pCREB with a peak expression at 3 h (Fig. 7H). The ATP- and FLX-evoked increase in pCREB in astrocytes was individually inhibited by suramin, NF-340 and MRS1706, and co-treatment with suramin and MRS1706 showed a further inhibition of pCREB formation (Figs. 7I, J). In addition, a PKA inhibitor, H-89, inhibited both ATP- and FLX-evoked pCREB (Figs. 7I, J).

Astrocytes constitutively release ATP [36], which is degraded by membrane-associated NTPDases [73]. Thus, the balance between release and degradation of ATP greatly affect extracellular ATP concentrations [39]. FLX inhibited NTPDases [46]; therefore, the FLX-evoked *Bdnf* mRNA upregulation seen in the present study may occur by the inhibition of NTPDases, rather than the stimulation of ATP exocytosis by FLX. To address this, we treated astrocytes with ARL67156 (100 μM), a selective inhibitor of NTPDases, but did not observe the upregulation of *Bdnf* mRNA (121.6 ± 2.7% of non-treated control, *n* = 5), suggesting the mechanism of BDNF upregulation by FLX cannot be explained by the inhibitory effect of FLX on NTPDase.

### 3.7 FLX Increases VNUT Via PKA-Dependent Mechanisms

**Fig. 8:**
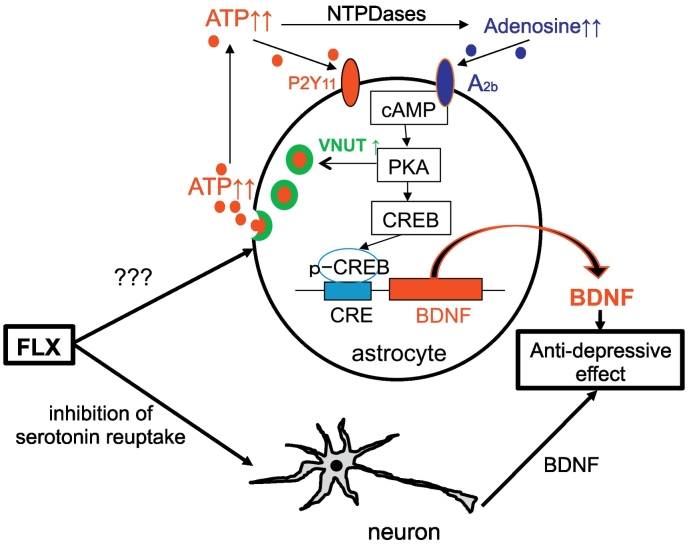
Schematic diagram of the mechanism involved in FLX-induced ATP release. FLX acts on neurons astrocytes to promote the release of ATP by exocytosis, which is dependent on VNUT. Released ATP and its metabolite adenosine respectively activate P2Y_11_ and A2b receptors expressed by astrocytes. The activation of both receptors results in an increase in cAMP, activation of PKA, and the induction of pCREB leading to an increase in the transcription of BDNF in astrocytes. Activated PKA also upregulates VNUT expression, leading to a feed-forward loop of FLX-evoked ATP release and BDNF increase.

Finally, we tested whether FLX affected VNUT expression in astrocytes. As shown in Fig. S8, treatment of astrocytes with FLX (30 μM) increased *Slc17a9* mRNA (encoding VNUT). The upregulation peaked at 12 h and lasted at least 24 h after FLX treatment. The increase in *Slc17a9* mRNA was abolished by H-89 (Fig. S8B), suggesting the involvement of PKA in *Slc17a9* mRNA upregulation. FLX-evoked ATP release peaked at 5 h (Fig. 1A) and lasted at least 10 h after FLX stimulation. Because FLX-evoked ATP release preceded the FLX-evoked upregulation of VNUT, this suggests that FLX stimulates ATP exocytosis via a VNUT-dependent mechanism, and the released ATP and its metabolite adenosine act on P2Y_11_ and A2b receptors, respectively, thereby causing the PKA-dependent upregulation of VNUT (Fig. 8). Such a feed-forward mechanism may affect ATP release and BDNF increase when FLX is administered chronically.

We also tested whether other psychotropic drugs affected VNUT expression. The SSRI-type antidepressants paroxetine and fluvoxamine upregulated *Slc17a9* mRNA in astrocytes, but the tetracyclic antidepressant mianserin did not (Fig. S8C). An antipsychotic drug haloperidol also had no effect (data not shown). Thus, there is a close correlation between *Bdnf* and *Slc17a9* upregulation by FLX and paroxetine, but not by mianserin (Fig. 4D).

## 4 Discussion

In general, major depression is thought to be caused by the dysfunction of monoaminergic neurons, because a number of antidepressants exert their primary biochemical effects by inhibiting the reuptake of 5-HT and/or noradrenaline [15]. Thus, antidepressants are believed to act on neurons especially monoaminergic neurons. SSRIs are the most commonly prescribed drugs for the treatment of depression, and are also thought to inhibit 5-HT reuptake in neurons. In addition, SSRIs also increase BDNF and neurogenesis [72], and these effects on neurons might contribute to their therapeutic effect. We demonstrated that astrocytes have a pivotal role in mediating the therapeutic effect of FLX (Fig. 2, Fig. 3). Few studies have shown that astrocytes are involved in the pathogenesis of depression (reviewed by [27]). For example, loss of glia but not neurons was sufficient to induce depressive-like behavior in rats [2], and FLX counteracted astrocytic cell loss in an animal model of depression [17]. Furthermore, the anti-depressant-like effects of imipramine were abolished when astrocytic function in the hippocampus was inhibited by fluorocitrate [31]. All these findings strongly suggest that astrocytic dysfunctions correlate with the pathogenesis of depression, and that anti-depressants might counteract these dysfunctions. However, these reports only reported a correlation between astrocytic functions and depressive behaviors or depression-related molecules, and did not show causality between them. A causal relationship as well as molecular mechanisms between astrocytes and depression is a still matter of debate. A recent report by Cao et al. reported a correlation between decreased extracellular ATP and depressive behavior, whereby (1) extracellular ATP concentrations in hippocampal astrocytes was low in depressive mice, and (2) when ATP was administered to mice, the repressive behavior was restored [6]. Thus, there seems to be a causal relationship between decreased extracellular ATP and depressive behavior, suggesting ATP might be an astrocytic molecule that controls depressive behavior. In the present study, we demonstrated that FLX, a SSRI antidepressant, increased extracellular ATP from hippocampal astrocytes by a VNUT-dependent mechanism. In addition, and most importantly, the FLX-induced anti-depressive effect was dependent on astrocytic VNUT (Fig. 3). A decrease or increase in VNUT in astrocytes decreased or increased the FLX-induced anti-depressive effects, respectively. Previous studies reported that FLX acted on neurons to mediate its therapeutic effects. In contrast, astrocytes have received limited attention as a therapeutic target of anti-depressants. Therefore, this study emphasizes that in addition to neurons, astrocytes also respond to FLX or anti-depressants, and contribute to its therapeutic effects. These findings strongly suggest that astrocytes might be a potential target for anti-depressants.

As described in the introduction, astrocytes possess multiple pathways for the release of ATP, including diffusible release from connexin hemi-channels [16], pannexin hemi-channels [61], P2X_7_ receptor channels, maxi-anion channels [40, 60], and exocytic release [24, 38, 44]. We previously showed that microglia, another type of glial cell, released ATP by exocytosis dependent upon VNUT [29]. In the present study, we clearly showed FLX increased ATP release from astrocytes by exocytosis because it was inhibited by bafilomycin A, BTX or the deletion of VNUT, but not by CBX (Fig. 1B, C). We did not determine how FLX stimulates ATP exocytosis from astrocytes, but there seems to be at least two distinct mechanisms: (1) the direct stimulation of VNUT-dependent ATP exocytosis (which we have not clarified but might be independent of 5-HT-mediated signals (Fig. S5E)); and (2) the upregulation of VNUT in astrocytes, based on our findings that released ATP activated P2Y_11_ receptors and its metabolite adenosine activated A2b receptors, upregulating VNUT in a PKA-dependent feed-forward mechanism. Furthermore, FLX upregulated VNUT, which was inhibited by the PKA inhibitor, H-89 (Fig. S8B). Based on differences in the time-course of FLX-evoked ATP release (Fig. 1A) and FLX-evoked VNUT-upregulation (Fig. S8A), events (1) and (2) probably occur separately. In addition to the inhibition of 5-HT uptake in neurons, FLX or other SSRIs have several other pharmacological functions. Tricyclic antidepressants [59] and SSRIs [43] were reported to inhibit Kir4.1 channels, an astrocyte-specific inwardly rectifying K channel. FLX inhibited Kir4.1 in astrocytes with an IC50 value of approximately 15 μM, similar to the ED50 value for FLX-evoked BDNF production in astrocytes in the present study (Fig. 4A). It is interesting that tricyclic anti-depressants and SSRIs inhibited Kir4.1 [43, 59] and produced BDNF in astrocytes (Fig. 4), but mianserin, a tetracyclic antidepressant, did not inhibit Kir4.1 [43] or produce BDNF in astrocytes (Fig. 4D). These similarities are interesting but we must await further experiments to clarify the involvement of Kir4.1 in the FLX-evoked ATP release in astrocytes.

How decreased extracellular ATP causes depressive effects, or how increased ATP mediates anti-depressive effects remain unknown. Cao et al. showed that astrocytic ATP acts on neuronal P2X receptors to mediate its therapeutic effects. However, detailed mechanisms have not been clarified. In the present study, we showed that FLX increased BDNF in astrocytes, which was ATP- and adenosine-dependent. BDNF has received increasing attention as a therapeutic target for depression because BDNF levels were reduced in mood disorders and preclinical depression models [33, 56], chronic treatment with anti-depressants increased brain BDNF gene expression and signaling [9], treatment with anti-depressants increased BDNF in serum in patients [53], and an infusion of BDNF into the midbrain [55] or hippocampus [54] produced anti-depressant-like effects in animal models of depression. In addition, patients with depression had SNPs of BDNF (X Jiang et al., 2005; Licinio et al., 2009). All these findings strongly suggest SSRIs might control BDNF-mediated signals, thereby leading to their therapeutic effects. Furthermore, all these reports showed the importance of neuronal BDNF. Therefore, the upregulation of BDNF by FLX-evoked ATP in astrocytes seen in the present study indicates it might mediate astrocyte-related anti-depressive effects. Anti-depressants including SSRIs were reported to increase BDNF in neurons [42]. In the present study, the chronic administration of FLX increased BDNF in hippocampal neurons, but this increase was greater in astrocytes (Fig. 5). Recent reports showed that BDNF expression in cultured astrocytes under several situations was upregulated by anti-depressants [1]. In this study, we demonstrated for the first time that BDNF was strongly upregulated in hippocampal astrocytes in chronically FLX-treated mice in vivo. The source of BDNF was reported to be mainly from neurons, and possibly from microglia in the CNS. Astrocytes have received limited attention as a source of BDNF because BDNF is expressed at low amounts in astrocytes of the normal adult brain. However, upon stimulation with FLX, astrocytes dramatically increased BDNF production from astrocytes in vitro (Fig. 4) and in vivo (Fig. 5). The brain contains higher numbers of astrocytes compared with neurons suggesting astrocytes might be a more important source of BDNF than neurons when exposed to FLX or anti-depressants.

What is the mechanism(s) underlying the ATP-mediated BDNF production in astrocytes? In neurons, SSRIs increase extracellular 5-HT, upregulating neuronal BDNF in a 5-HT receptor-dependent manner [4]. However, unlike neurons, the FLX-evoked BNDF increase in astrocytes was independent of 5-HT, but was dependent on P2 and P1 receptors. Released ATP acts on several types of P2 receptors [48], and is immediately metabolized into adenosine by NTPDases and 5′-nucleotidases [73]. We showed that ATP and adenosine act on P2Y_11_ and A2b receptors, respectively, and upregulate BDNF via cAMP/PKA/pCREB-dependent pathways (Fig. 6, Fig. 7, S5, S6). Some studies have reported A2b receptors in astrocytes [47, 69], and few studies have reported P2Y_11_ receptors in astrocytes [3]. Astrocytic P2Y_11_ receptors were functional and were inhibited by NF340 or MRS1706 (Fig. 6A). In addition, cultured astrocytes expressed anti-P2Y_11_ receptor antibody-positive signals, which disappeared when the antibody was absorbed by its antigen-peptide (Fig. S2B). Thus, both P2Y_11_ and A2b receptors appear to be present and functional in astrocytes. Both receptors are Gs-coupled GPCR, and their activation results in cAMP/PKA pathway signaling in astrocytes. We demonstrated that the PKA-dependent formation of pCREB, a well-known transcription factor [19], is a key event that upregulates *Bdnf* mRNA in astrocytes. The inhibitory effect of FLX-evoked BDNF production and formation of pCREB by suramin or MRS1706 was accentuated by co-application of both antagonists, suggesting that at least in part, P2Y_11_ and A2b receptors might contribute to these events independently.

FLX induced a marked increase in astrocytic BDNF in the hippocampus, but only a small increase in the cortex. Thus, the effect of FLX on BDNF upregulation seems to be dependent on the brain region. We must await further investigation to clarify why such a difference occurs. However, this region-dependent upregulation of astrocytic BDNF by FLX may reveal new findings as to how and where anti-depressants mediate their therapeutic effects.

In conclusion, we demonstrated that the anti-depressant FLX acted on astrocytes, and mediated its therapeutic effects by facilitating VNUT-dependent ATP exocytosis. Decreased or increased VNUT in astrocytes resulted in decreased and increased FLX-evoked anti-depressive effects, respectively, suggesting astrocytic ATP exocytosis via VNUT plays a pivotal role in modulating the therapeutic effect of FLX. The upregulation of BDNF in astrocytes might be the most likely event in FLX-evoked ATP-mediated anti-depressive effects. In addition to FLX, other anti-depressants also increased VNUT and BDNF in astrocytes, suggesting the astrocytic regulation seen in the present study might be a common pharmacological profile for anti-depressants.

## Appendix A Supplementary data

Supplementary material Image 1
