# Supplementary material for: Anti-Depressant Fluoxetine Reveals its Therapeutic Effect Via Astrocytes
Source: eBioMedicine. 2018 Jun 8;32:72–83. doi: 10.1016/j.ebiom.2018.05.036 (PMC6020856; doi:10.1016/j.ebiom.2018.05.036)
Supplement: Supplementary file 1 — Supplementary material [file mmc1.pdf]

SUPPLEMENTAL INFORMATION

Supplemental Figures

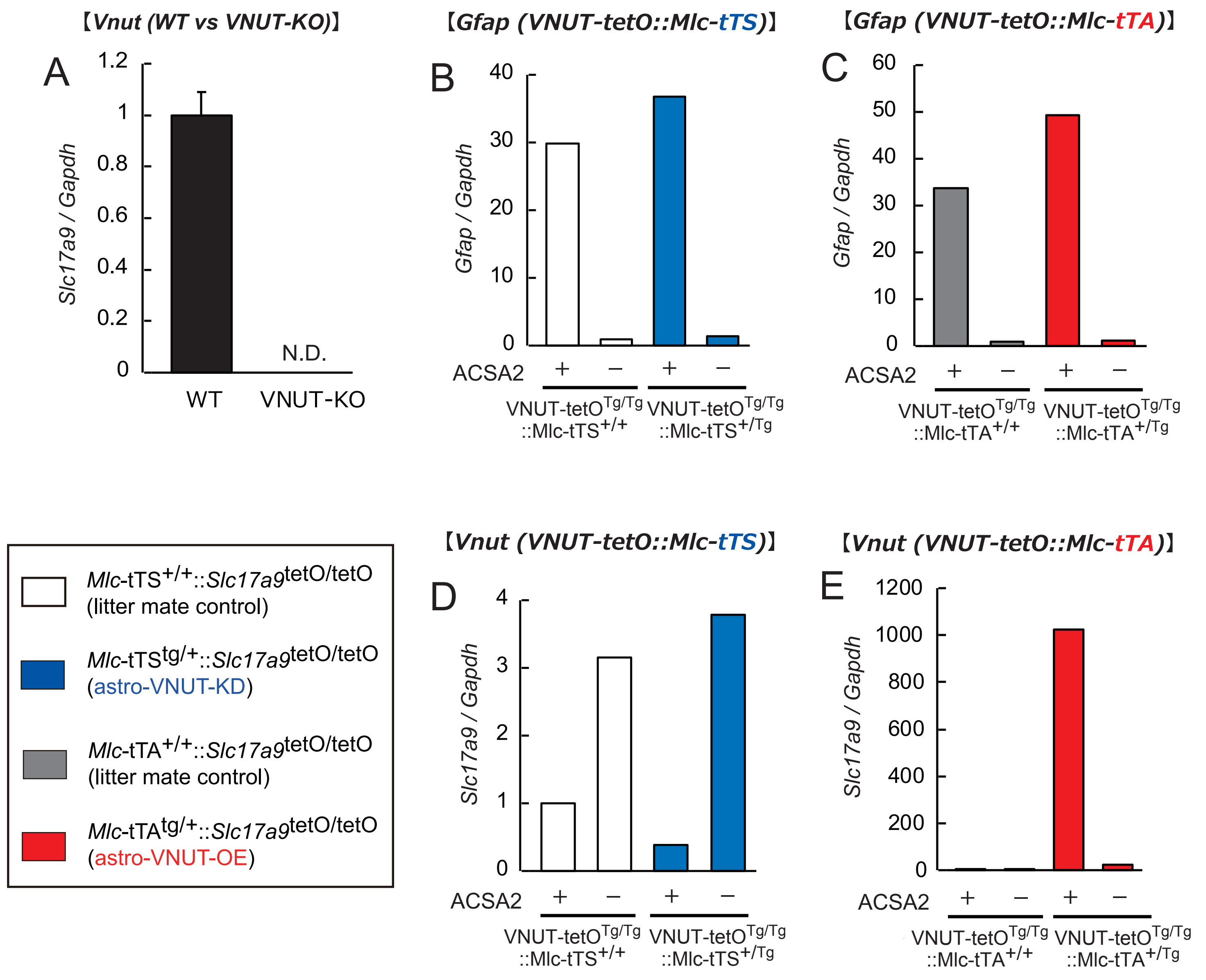

Figure S1. Conventional knockout and astrocyte-specific overexpression or knockdown of VNUT.

(A) *Slc17a9* mRNA (encoding VNUT) levels were evaluated by quantitative PCR in the hippocampal tissues of wild-type (WT) or VNUT-KO mice. (B-E) Astrocytes were purified from the hippocampal tissues of adult astro-VNUT-OE (*vnut-tetO*::*Mlc-tTA*) and astro-VNUT-KD (*vnut-tetO*::*Mlc-tTS*) mice by magnetic-activated cell sorting (MACS) with astrocyte cell surface antigen-2 (ACSA-2) antibodies. Total RNA was extracted and *Slc17a9* and *glial fibrillary acidic protein (gfap)* mRNA levels were evaluated by qPCR from ACSA-2-positive astrocytes and ACSA-2-negative cells. (B and C) Purification of astrocytes from astro-VNUT-OE and astro-VNUT-KD mice. (D and E) Astrocytes purified from astro-VNUT-KD and astro-VNUT-OE mice showed decreased and increased *Slc17a9* mRNA levels, respectively. ACSA-2-negative fractions showed no differences in *Slc17a9* mRNA levels. Values were normalized to WT mice (A), ACSA-2-negative fractions from *Mlc-tTS*$^{+/+}$::*Slc17a9*$^{tetO/tetO}$ mice (B), ACSA-2-negative fractions from *Mlc-tTA*$^{+/+}$::*Slc17a9*$^{tetO/tetO}$ mice (C and E) and ACSA-2-positive fractions from *Mlc-tTS*$^{+/+}$::*Slc17a9*$^{tetO/tetO}$ mice (D).

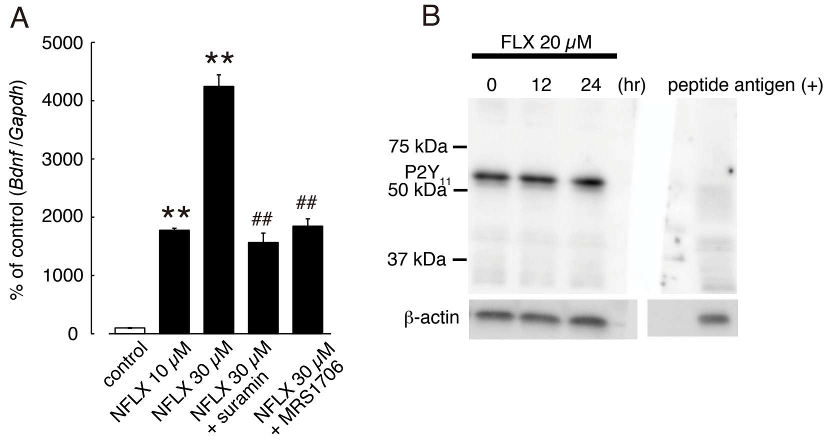

Figure S2. Norfluoxetine-evoked increase in *Bdnf* mRNA and the involvement of P2 and P1 receptors

(A) Norfluoxetine (NFLX), an active metabolite of FLX, elicited an increase in *Bdnf* mRNA in astrocytes in a concentration dependent manner (from 10 to 30 μM). Hippocampal astrocytes were treated with NFLX or PBS (control) for 12 hr with or without P2 or A2b receptor antagonists (100 μM suramin or 1 μM MRS1706). Values were normalized to PBS-treated controls and shown as a % of the control. Data show the mean ± SEM (n=4). ***p* < 0.01 vs. PBS-treated control. ##*p* < 0.01 vs. NFLX (30 μM) alone. (B) Western blot analysis of the expression of $P2Y_{11}$ receptors in primary cultures of hippocampal astrocytes, showing the effect of FLX. Cells were treated with FLX (20 μM), incubated for 0, 12 or 24 hr, and probed with anti-$P2Y_{11}$ receptor antibody. In the right lane, the $P2Y_{11}$ receptor-positive band disappeared when $P2Y_{11}$ receptor control peptide antigen was added and the anti-$P2Y_{11}$ receptor antibody was absorbed. These data represent at least three independent experiments.

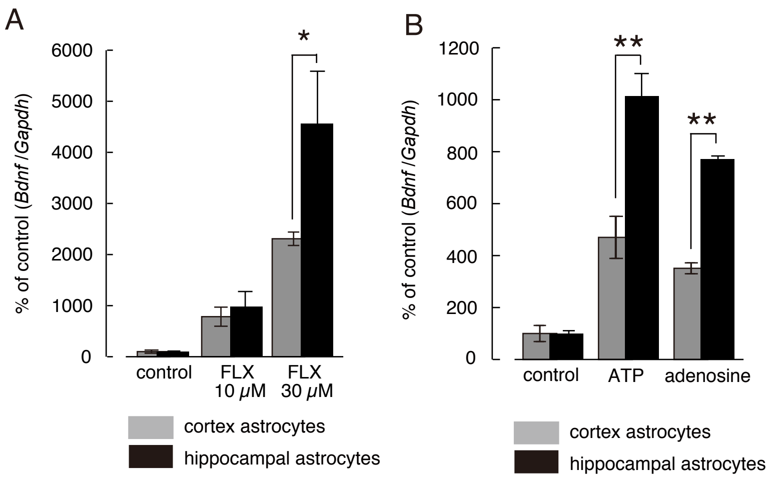

Figure S3. Comparison of FLX-, ATP- and adenosine-induced *Bdnf* mRNA expression levels between rat cortical astrocytes and hippocampal astrocytes

Primary cultures of hippocampal and cortical astrocytes were treated with (A) FLX (10 or 30 μM) for 12 hr and *Bdnf* mRNA was analyzed by RT-PCR (*$p < 0.05$) or (B) ATP (100 μM) or adenosine (100 μM) for 1 hr and *Bdnf* mRNA was analyzed by RT-PCR (**$p < 0.01$). Values were normalized to the PBS control and shown as a % of the control value. Data show the mean ± SEM of three independent experiments.

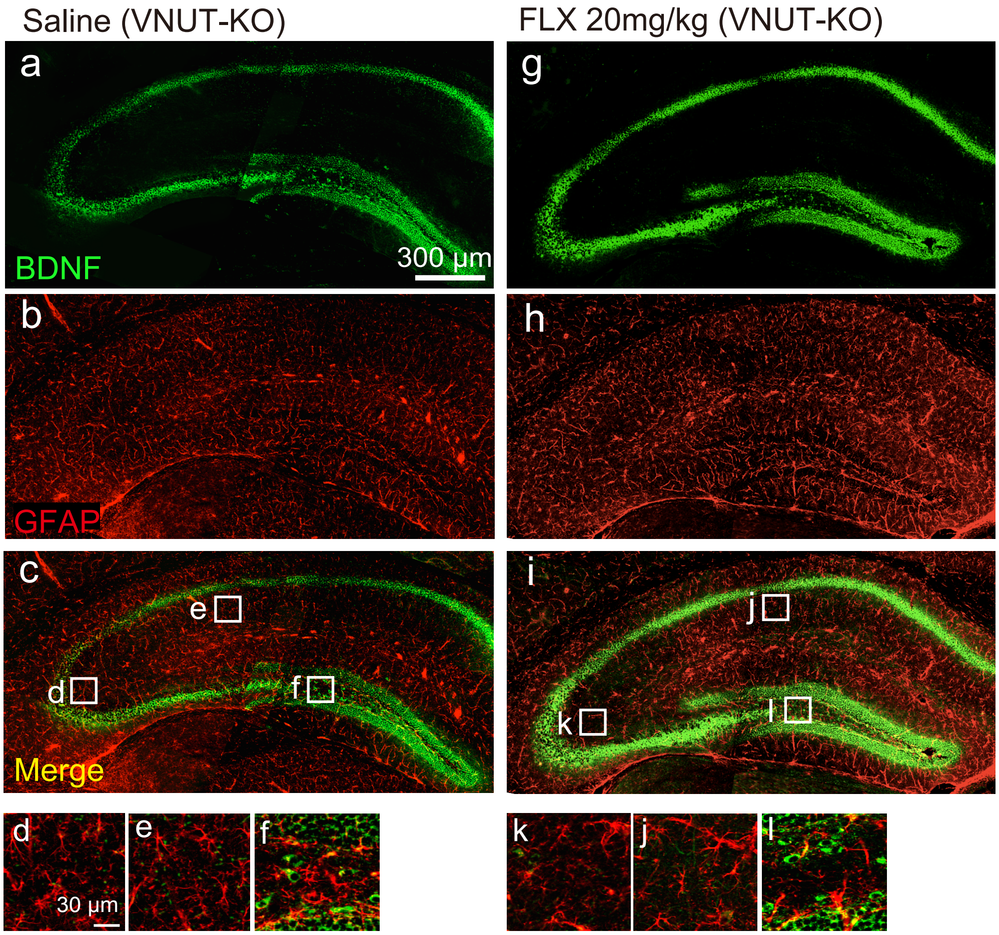

Figure S4. BDNF upregulation in hippocampal astrocytes by the chronic administration of FLX is absent in VNUT-KO mice

Double immunostaining of hippocampal sections from VNUT-KO mice with anti-BDNF (green) and anti-GFAP (red) antibodies as per Figure 5. ***a-c*** are images of saline-administered mice. ***g-i*** are FLX (20 mg/kg for 21 days, p.o.)-administered mice. ***d-f*** and ***k-l*** are magnifications of the insets shown in c and i, respectively. In VNUT-KO mice, BDNF-positive signals are less elevated in GFAP-positive astrocytes after the chronic administration of FLX (Figure ***i-l***), whereas BDNF immunoreactivities are dramatically increased in GFAP-positive astrocytes in FLX-treated WT mice (see Figure 5 ***k-n***). Scale bars: a, 300 μM; d, 30 μM. These representative data are from at least 3 independent experiments where similar results were obtained.

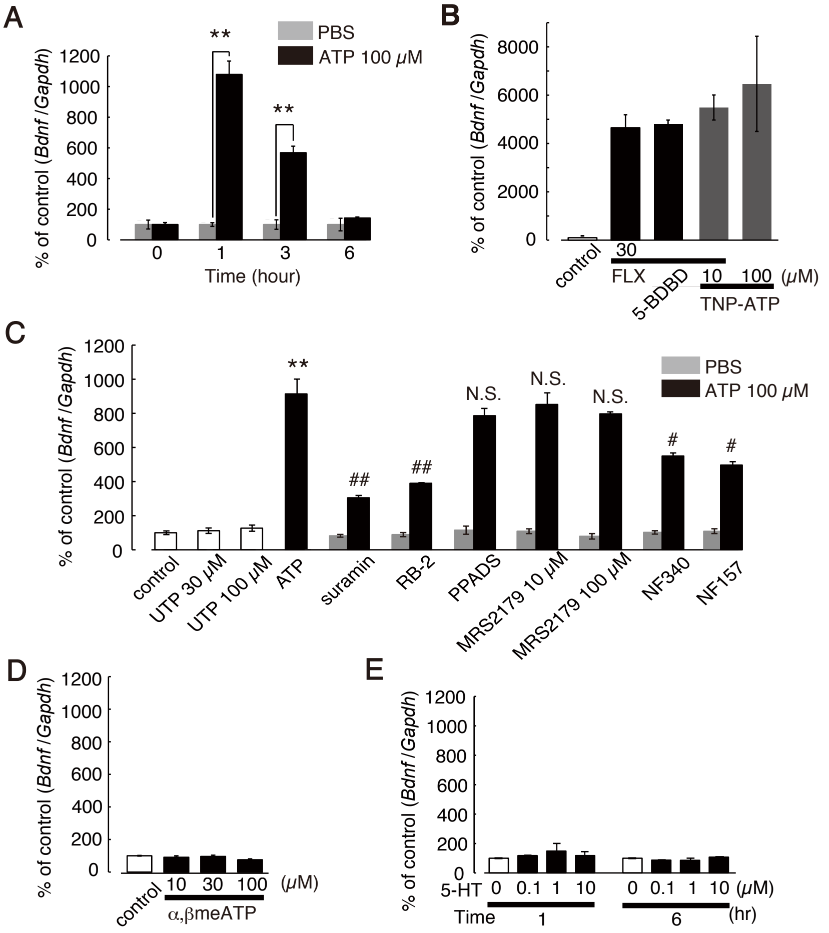

Figure S5. Pharmacological characterization of increased *Bdnf* mRNA expression in hippocampal astrocytes by ATP, ATP analogues and 5-HT

(A) Time-course of the ATP-evoked increase of *Bdnf* mRNA in hippocampal astrocytes. Cells were incubated with ATP (100 μM) or PBS for 1, 3 or 6 hr, and then *Bdnf* mRNA was assessed at each time point. Gray and black columns indicate the PBS-treated control and ATP (100 μM)-treated groups, respectively. $^{**}p < 0.01$ vs. PBS control. (B) Effect of P2X receptor antagonist TNP-ATP (0.1 or 1 μM) or 5-BDBD (10 μM) on the FLX-evoked increase of *Bdnf* mRNA in hippocampal astrocytes. Cells were treated with each concentrations of TNP-ATP or 5-BDBD for 30 min before and during FLX stimulation for 12 hr. (C) Pharmacological identification of P2 receptors responsible for the ATP-evoked increase of *Bdnf* mRNA in hippocampal astrocytes. Cells were treated with various antagonists including wide-range P2 receptor antagonist suramin (100 μM), P2Y receptor antagonist RB-2 (10 μM), P2X receptor antagonist PPADS (100 μM), $P2Y_1$ receptor antagonist MRS2179 (10 or 100 μM), or $P2Y_{11}$ receptor antagonists NF340 (30 μM) and NF157 (10 μM) for 30 min before and during ATP stimulation (1 hr). Cells were stimulated with a $P2Y_2$, $P2Y_4$ and $P2Y_6$ receptor agonist UTP (30 and 100 μM) (white columns) for 1 hr. Gray and black columns indicate PBS-treated control and ATP (100 μM)-treated groups. $^{**}p < 0.01$ vs. PBS-treated control, $^{\#}p < 0.05$, $^{\#\#}p < 0.01$ vs. ATP-treated group, N.S., not significant. (D) Activation of P2X receptors by α,βmeATP had no effect on *Bdnf* mRNA expression in hippocampal astrocytes. Cells were treated with various concentrations of α,βmeATP for 1 hr. (E) 5-HT did not increase *Bdnf* mRNA expression in hippocampal astrocytes. Cells were incubated with various concentrations of 5-HT (0.1–10 μM) for 1 or 6 hr. *Bdnf* mRNA expressions were assessed by quantitative RT-PCR. Values were normalized to the PBS-treated control and shown as a % of the control value. Data are the mean ± SEM of at least three independent experiments.

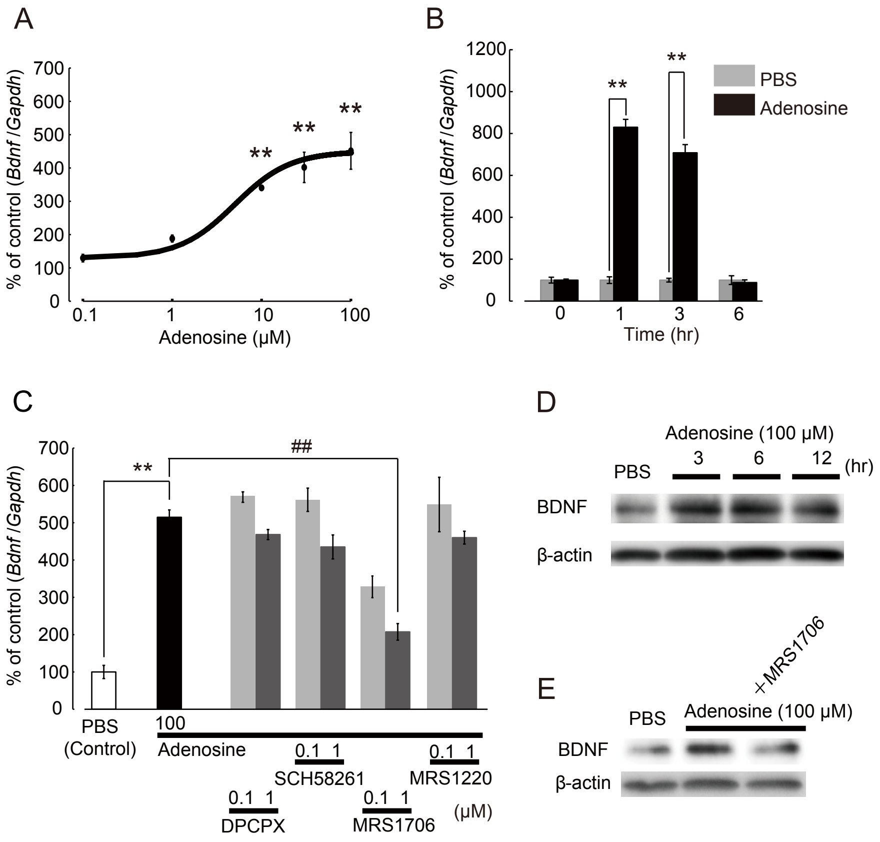

Figure S6. Pharmacological characterization of the adenosine-evoked increase of *Bdnf* mRNA in hippocampal astrocytes

(A) Adenosine increases *Bdnf* mRNA dose-dependently in hippocampal astrocytes. Cells were incubated with various concentrations of adenosine for 1 hr in serum-free culture medium (0.1–100 μM). ***p* < 0.01 vs. PBS (adenosine free)-treated control. (B) Time-course of the adenosine-evoked increase in *Bdnf* mRNA in hippocampal astrocytes. Cells were treated with adenosine (100 μM) for 1, 3 or 6 hr. Gray and black columns indicate PBS-treated control and adenosine (100 μM)-treated groups, respectively. ***p* < 0.01 vs. PBS-treated control. (C) Pharmacological identification of P1 receptors responsible for the adenosine-evoked increase of *Bdnf* mRNA in hippocampal astrocytes. Cells were treated with A1 receptor antagonist DPCPX, A2a receptor antagonist SCH58261, A2b receptor antagonist MRS1706 or A3 receptor antagonist MRS1220 for 30 min before and during adenosine (100 μM) stimulation (1 hr). Gray and black columns indicate adenosine plus various concentrations (0.1 and 1 μM) of antagonists and adenosine (100 μM) alone, respectively. ***p* < 0.01 and ##*p* < 0.01 vs. adenosine alone. Values were normalized to PBS-treated control expression and shown as the mean ± SEM (n=4). (D) Time-course of the adenosine-evoked increase of BDNF protein in hippocampal astrocytes. Cells were stimulated with 100 μM adenosine for 3, 6, and 12 hr, and then western blot analysis was performed. (E) Effect of MRS1706 (1 μM) on the adenosine-evoked increase of BDNF in hippocampus astrocytes. Cells were treated with MRS1706 (1 μM) for 30 min before and during adenosine (100 μM) stimulation. BDNF protein levels were assessed by western blot analysis 6 hr after adenosine-treatment.

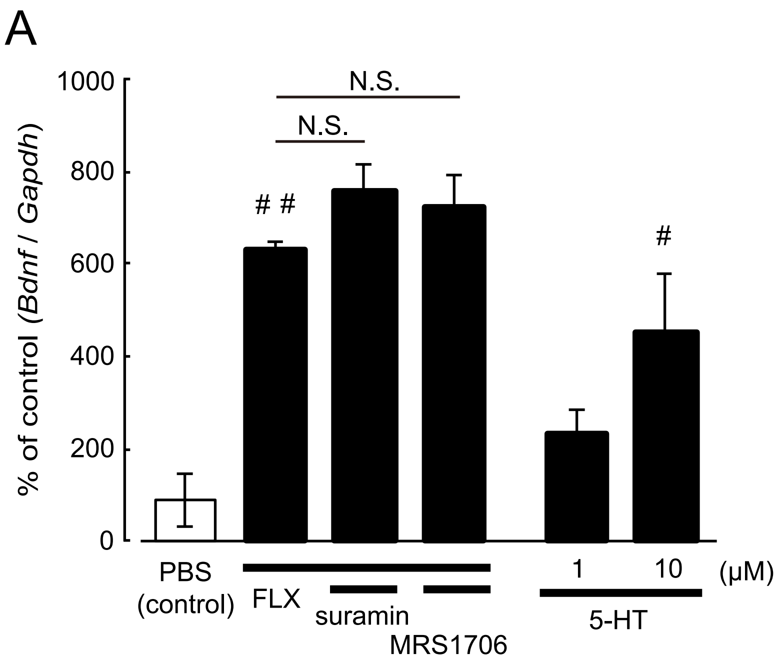

Figure S7. Pharmacological characterization of increased *Bdnf* mRNA expression in hippocampal neurons by FLX and 5-HT

Hippocampal neurons were treated with each receptor antagonist (100 μM **s**uramin and 1 μM MRS1706) and stimulated by FLX (10 μM) or treated with 1 or 10 μM 5-HT. After stimulation for 12 hr, *Bdnf* mRNA was analyzed by RT-PCR. $^{\#\#}p < 0.01$ or $^{\#}p < 0.05$ vs. PBS-treated control, respectively. N.S., not significant. Values are normalized to the PBS-treated control and are shown as a % of the control value. Data show the mean ± SEM of three independent experiments.

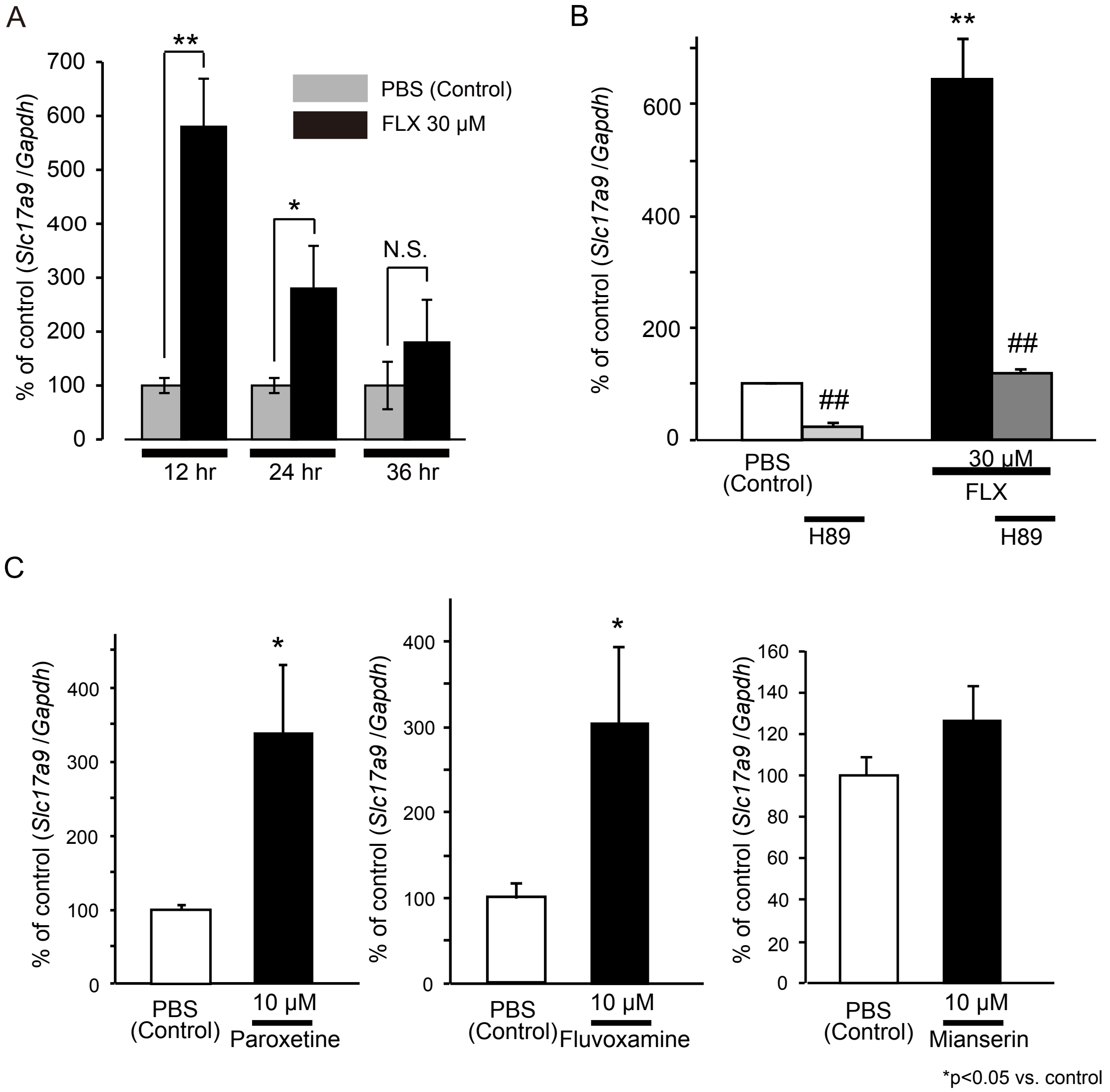

Figure S8. FLX and other anti-depressants increase *Slc17a9* mRNA expression in hippocampal astrocytes
(A) Time-course of the FLX-evoked increase of *Slc17a9* mRNA expression in astrocytes. Cells were incubated with FLX (30 μM) or PBS for 12, 24 or 36 hr in serum-free culture medium. Gray and black columns indicate PBS-treated control and FLX-treated groups, respectively. *$p < 0.05$, **$p < 0.01$ vs. PBS control, N.S., not significant. (B) Effect of H-89 on the FLX-evoked increase of *Slc17a9* mRNA expression in hippocampal astrocytes. Cells were treated with the PKA antagonist H-89 (20 μM) for 30 min before and during FLX (30 μM) stimulation for 12 hr. **$p < 0.01$ vs. PBS-treated control. $^{##}p < 0.01$ vs. PBS alone or FLX alone, respectively. (C) Effects of other antidepressants on the increase of *Slc17a9* mRNA expression in hippocampal astrocytes. Cells were treated with various anti-depressants (10 μM Paroxetine, 10 μM Fluvoxamine or 10 μM Mianserin) for 12 hr, and then *Slc17a9* mRNA expression was assessed by quantitative RT-PCR. *$p < 0.05$ vs. PBS-treated control. Values were normalized to the PBS-treated control and are shown as a % of the control value. Data are the mean ± SEM (n=3–7).
